# ﻿New species of *Nigrobaetis* from Southeast Asia (Ephemeroptera, Baetidae)

**DOI:** 10.3897/zookeys.1166.102941

**Published:** 2023-06-09

**Authors:** Thomas Kaltenbach, Jean-Luc Gattolliat

**Affiliations:** 1 Muséum cantonal des Sciences Naturelles, Département de Zoologie, Palais de Rumine, Place Riponne 6, CH-1005 Lausanne, Switzerland Muséum cantonal des Sciences Naturelles Lausanne Switzerland; 2 University of Lausanne (UNIL), Department of Ecology and Evolution, CH-1015 Lausanne, Switzerland University of Lausanne Lausanne Switzerland

**Keywords:** COI, eggs, Indonesia, mayflies, Philippines, systematics

## Abstract

Based on larvae collected in the Philippines, Borneo (Kalimantan), Sumba, and Sumatra, the presence of the genus *Nigrobaetis* in the Philippines and Indonesia is reported for the first time. Six new species are described and illustrated, two from the Philippines and four from Indonesia. A larval key to all *Nigrobaetis* species of the Philippines, Indonesia and neighbouring continental Southeast Asia is provided, morphological differences to the species of Taiwan are discussed as well. The eggs of three new species are described, and the morphology of the eggs of *Nigrobaetis* is briefly discussed.

## ﻿Introduction

*Nigrobaetis* Novikova & Kluge, 1987 is a genus of Baetidae, which is the most diverse family of mayflies in number of genera and species (Sartori and Brittain 2015; Jacobus et al. 2019). *Nigrobaetis* has a wide distribution across the Afrotropical (six species), Palearctic (15 species) and Oriental regions (12 species), being absent from the Nearctic, the Neotropics region and Australasia (Barber-James et al. 2013).

They are mostly small species with a body length of 3–5 mm for the mature larvae, and present the following characters: larval antennae standing closely together, with a longitudinal keel between them (Lugo-Ortiz and de Moor 2000: fig. 1); right mandible with row of long, setae-like processes between prostheca and mola (Fig. 10c); left mandible usually with row of minute denticles between prostheca and mola (Fig. 2d); rarely absent or with tuft of medium, setae-like processes (Figs 10d, 16d); labial palp segment II without distomedial protuberance, segment III generally roughly quadrangular, apically truncated (Fig. 4a, c); subimaginal gonostyli developing under cuticle of male last instar larvae folded in the *Nigrobaetis*-type (Figs 6c, 26c; Kluge 2004: fig. 29G; Kluge 2022).

From the Oriental region, *Nigrobaetis* species were described from continental Southeast Asia (Müller-Liebenau 1984), from Taiwan (Müller-Liebenau 1985; Kang et al. 1994; Kang and Yang 1996), and from India (Kubendran et al. 2015; Sivaruban et al. 2022). The important diversity reported from Taiwan (six species), points into the direction that we may expect many more species in other parts of the generally species-rich Southeast Asia. In comparison, the Afrotropical diversity is much lower with a single species widely distributed in continental Africa, and three species endemic to a single island (Anjouan, Madagascar, or la Réunion; Lugo-Ortiz and de Moor 2000; Gattolliat 2004; Kaltenbach et al. 2021). *Nigrobaetis* presently encompasses 33 species, 27 of them being described in the last forty years. In this study, we report the genus *Nigrobaetis* for the first time from the Philippines and Indonesia. We describe and illustrate six new species based on larvae and partly also on eggs.

Indonesia is an immense archipelago of more than 18.000 islands extending over a huge area from 95°E to 141°E and from 6°N to 11°S. It is one of the most biologically rich countries in the world. The high levels of species richness and endemism are mainly explained by a geological history that brought together two different biological realms (Oriental realm and Australasian realm), separated by a transitional region (Wallacea) (Hall 2010; Kingston 2010). The archipelago includes the main part of the Sundaland Biodiversity Hotspot, influenced by a dynamic and highly complex geophysical history including changing climates, fluctuating sea levels, volcanism, and orogenic activity with subsequent erosion (Quek 2010).

Similarly, the Philippines are a complex archipelago with more than 7100 islands, spanning the Asian-Australian faunal zone interface directly at the Wallace Line. The Huxley Line is dividing the country into Palawan and associated islands, the presumed former land-bridge to northern Borneo, and the truly oceanic portions of the Philippines. It has an extraordinary biodiversity, presumably supported by ancient land mass movements, environmental gradients along steep volcanic slopes and alterations of connectivity between neighbouring islands induced by changing sea levels (Brown and Diesmos 2010).

Despite many collection efforts and progress done in the past decade in Southeast Asia, the Baetidae and Ephemeroptera in general still remain poorly known. During this study, we identified several additional new species of *Nigrobaetis* from both the Philippines and Indonesia, but refrained to describe them because of insufficient material (mostly one larva only). Therefore, we may expect a substantial amount of additional new species of *Nigrobaetis* with more collections in the future.

## ﻿Materials and methods

Specimens used in the study were obtained by kick-sampling and preserved in 70%–96% ethanol.

Eggs of three species were extracted from the abdomen of mature female larvae, and preserved in 96% alcohol before preparation (desiccation; application of a 15 nm layer of palladium) for SEM photos.

Dissection of larvae was done in Cellosolve (2-Ethoxyethanol) with subsequent mounting on slides with Euparal liquid, using an Olympus SZX7 stereomicroscope.

Photographs of larvae were taken using a Canon EOS 6D camera and processed with the programs Adobe Photoshop Lightroom (http://www.adobe.com) and Helicon Focus version 5.3 (http://www.heliconsoft.com). Photographs of larval parts on slides were taken with an Olympus BX43 microscope equipped with an Olympus SC 50 camera and the program Olympus CellSense v. 3.2. SEM pictures were taken using a FEI Quanta FEC 250 electron microscope (Thermo Fisher). All photographs were subsequently enhanced with Adobe Photoshop Elements 13.

The DNA of part of the specimens was extracted using non-destructive methods allowing subsequent morphological analysis (see Vuataz et al. 2011 for details). We amplified a 658 bp fragment of the mitochondrial gene cytochrome oxidase subunit 1 (COI) using the primers LCO 1490 and HCO 2198 (Folmer et al. 1994; see Kaltenbach and Gattolliat 2020 for details). Sequencing was done with Sanger’s method (Sanger et al. 1977). The genetic variability between specimens was estimated using Kimura-2-parameter distances (K2P, Kimura 1980), calculated with the program MEGA 7 (Kumar et al. 2016, http://www.megasoftware.net). Additionally, we downloaded a COI sequence of *N.minutus* (Müller-Liebenau, 1984) from GenBank. There were no other COI sequences of *Nigrobaetis* from Southeast Asia available on GenBank. GenBank accession numbers are given in Table 2.

The distribution maps were generated with the program SimpleMappr (https://simplemappr.net, Shorthouse 2010); the GPS coordinates of the sample locations are given in Table 1.

**Table 1.** GPS coordinates of sample locations.

**Species**	**Country**	**Location**	**GPS coordinates**
*Nigrobaetisplures* sp. nov.	Philippines	Leyte	10°01'07"N, 125°12'35"E
		Mindanao	09°03'33"N, 126°05'57"E
			09°11'34"N 125°36'34"E
			09°10'15"N, 125°40'55"E
		Camiguin	09°06'39"N, 124°43'45"E
*Nigrobaetispalawus* sp. nov.	Philippines	Palawan	10°23'35"N, 119°09'27"E
			10°22'40"N, 119°11'05"E
*Nigrobaetissumbensis* sp. nov.	Indonesia	Sumba	09°35'45"S, 119°20'25"E
*Nigrobaetissuma* sp. nov.	Indonesia	Sumatra: Marapi	00°2158"S, 100°33'18"E
			00°21'33"S, 100°30'42"E
			00°28'29"S, 100°22'08"E
		Sumatra: Sago	00°17'08"S, 100°41'13"E
			00°22'33"S, 100°39'33"E
			00°22'20"S, 100°41'45"E
		Sumatra: Singgalang	00°22'56"S, 100°22'42"E
			00°19'57"S, 100°19'19"E
			00°24'07"S, 100°16'44"E
			00°23'33"S, 100°16'34"E
		Sumatra: Talamau	00°00'60"N, 100°00'01"E
*Nigrobaetisborneus* sp. nov.	Indonesia	Borneo, Kalimantan	02°59'22"N, 116°30'46"E
*Nigrobaetiskaliman* sp. nov.	Indonesia	Borneo, Kalimantan	03°00'10"N, 116°32'24"E
			03°04'04"N, 116°30'26"E
			02°59'29"N, 116°33'29"E
			03°04'56"N, 116°30'58"E
			03°00'05"N, 116°30'48"E

**Table 2.** Interspecific (bold) and intraspecific genetic distance (COI; Kimura 2-parameter) of some *Nigrobaetis* species.

	**Species**	**Location**	**Specimens catalog** #	**GenBank** #	**1**	**2**	**3**	**4**	**5**	**6**	**7**
**1**	*N.plures* sp. nov.	Philippines: Leyte	GBIFCH00975650	OQ569761							
**2**	*N.suma* sp. nov.	Indonesia: Sumatra, Marapi	GBIFCH00422020	OQ569762	**0,22**						
**3**		Indonesia: Sumatra, Marapi	GBIFCH00422001	OQ569763	**0,22**	0,01					
**4**		Indonesia: Sumatra, Sago	GBIFCH00422027	OQ569764	**0,22**	0,02	0,01				
**5**		Indonesia: Sumatra, Sago	GBIFCH00421990	OQ569765	**0,22**	0,02	0,01	0,00			
**6**		Indonesia: Sumatra, Singgalang	GBIFCH00422033	OQ569766	**0,22**	0,00	0,01	0,02	0,02		
**7**		Indonesia: Sumatra, Talamau	GBIFCH00421981	OQ569767	**0,22**	0,02	0,01	0,00	0,00	0,02	
**8**	*N.minutus*	Thailand	n/a	HM417038.1	**0,23**	**0,22**	**0,21**	**0,23**	**0,23**	**0,22**	**0,23**

The dichotomous key was elaborated with the support of the program DKey version 1.3.0 (http://drawwing.org/dkey, Tofilski 2018).

The terminology follows Hubbard (1995) and Kluge (2004). The term “setae-like processes” is used for medium to long, thin (hair-like) processes, which are not articulated as real setae; they often occur between prostheca and mola of mandibles (usually referred to as setae), and sometimes distally on margin of labial palp segment III.

### ﻿Abbreviations

**AdMU** Ateneo de Manila University, Quezon City, Philippines;

**MZL** Muséum cantonal des Sciences Naturelles, Département de Zoologie, Lausanne (Switzerland);

**PNM**Museum of Natural History of the Philippine National Museum, Manila (Philippines);

**MZB**Museum Zoologicum Bogoriense (Indonesia).

## ﻿Results

### ﻿List of described species

Philippines

1. *N.plures* sp. nov.

2. *N.palawus* sp. nov.

#### ﻿Indonesia

3. *N.sumbensis* sp. nov.

4. *N.suma* sp. nov.

5. *N.borneus* sp. nov.

6. *N.kaliman* sp. nov.

##### ﻿ Nigrobaetis plures sp. nov.

Taxon classificationAnimaliaEphemeropteraBaetidae

AF734AF3-2E09-5244-98EE-2D31B54DEE04

https://zoobank.org/88034E97-CBC0-4831-863C-F56104AC0004

Figs 1 , 2 , 3 , 4 , 5 , 6 , 7 , 8

###### Differential diagnosis.

**Larva.** Following combination of characters: A) dorsal surface of labrum with submedian seta and two long, simple setae in submarginal position (Fig. 2b); B) right mandible: incisor with four denticles, kinetodontium with four denticles (Fig. 2c); C) left mandible: incisor with four or five denticles, kinetodontium with three denticles; margin between prostheca and mola with row of minute denticles (Fig. 2d); D) fore femur length ca. 3× maximum width, dorsal margin with 7–11 curved, spine-like setae (Fig. 5a); E) claw with 11 or 12 denticles (Fig. 5b); F) hind protoptera minute (Fig. 5d); G) tergalii on abdominal segments I–VII; H) paraproct with ca. six stout, large marginal spines (Fig. 6b); I) posterior margins of abdominal terga: I–III smooth, without spines; IV–V with rudimentary spines or smooth; VI–IX with triangular, sharply pointed spines (Fig. 6a).

###### Description.

**Figure 1.**
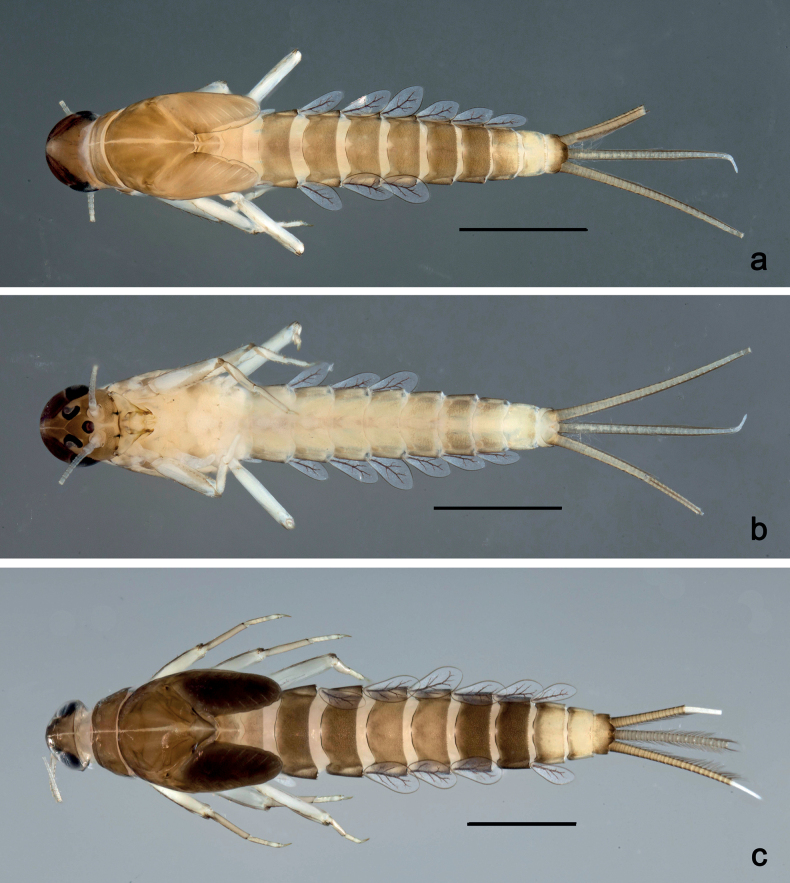
*Nigrobaetisplures* sp. nov., larva habitus **a** dorsal view (Leyte) **b** ventral view (Leyte) **c** dorsal view (Mindanao). Scale bars: 1 mm.

**Figure 2.**
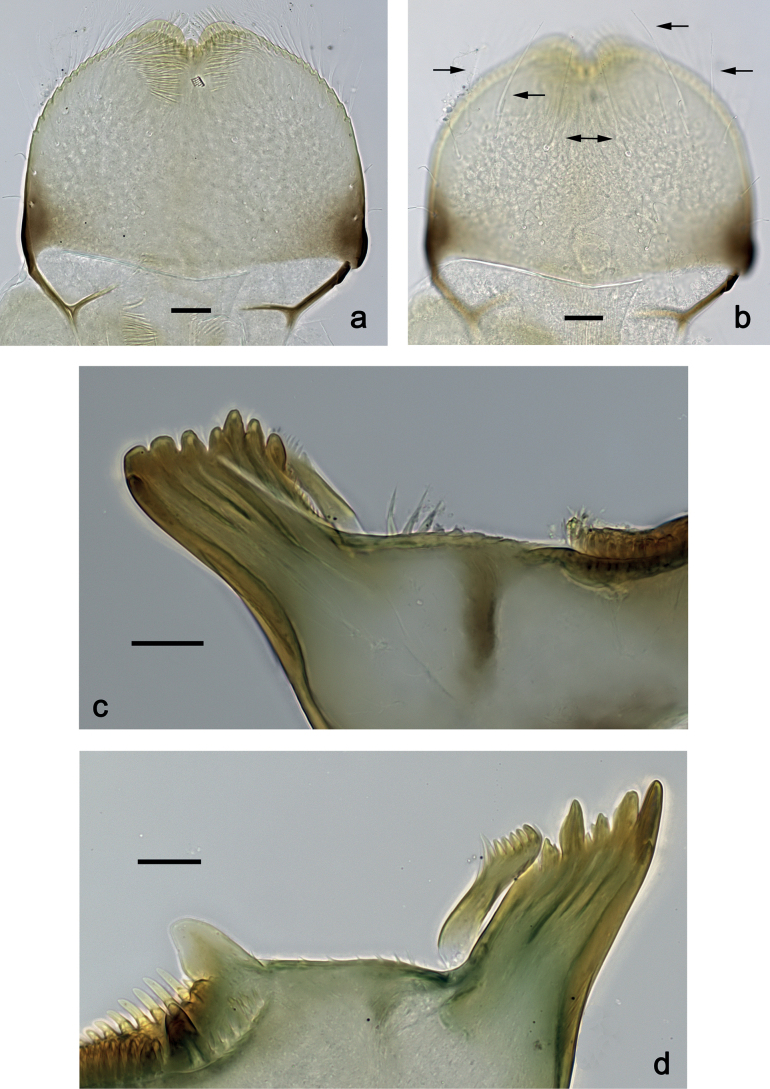
*Nigrobaetisplures* sp. nov., larva morphology **a** labrum, ventral focus **b** labrum, dorsal focus (arrows: submedian and submarginal setae) **c** right mandible **d** left mandible. Scale bars: 20 µm.

**Figure 3.**
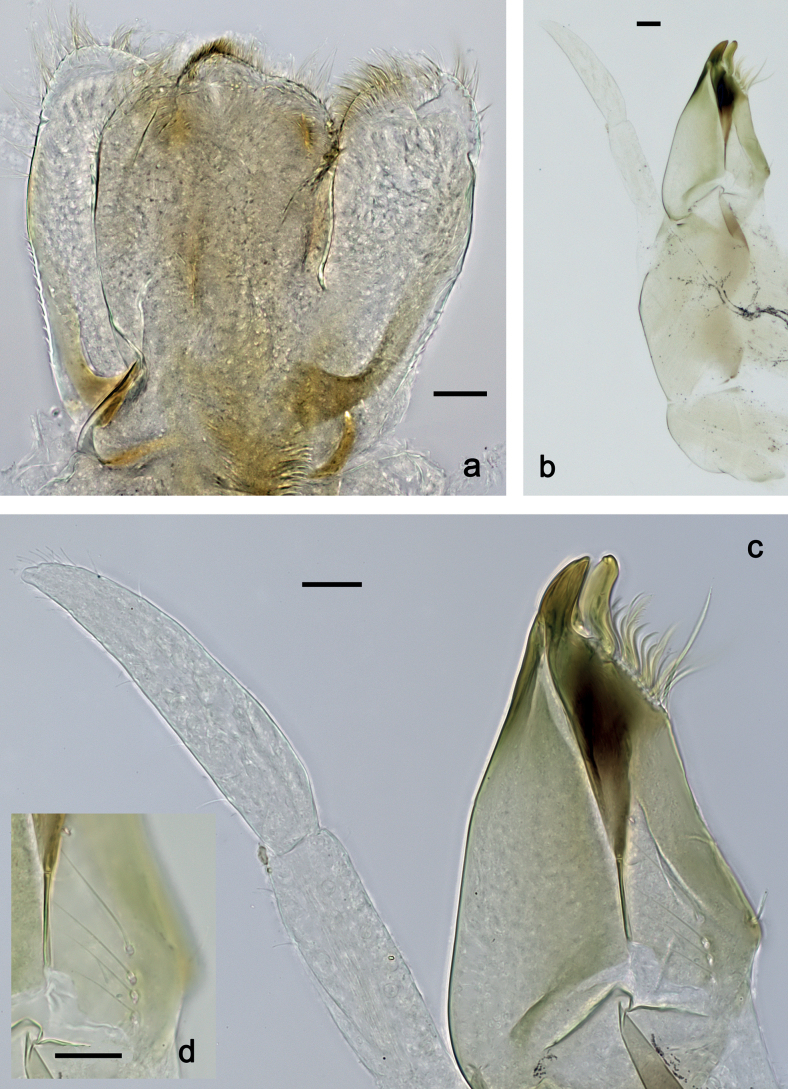
*Nigrobaetisplures* sp. nov., larva morphology **a** hypopharynx and superlinguae **b, c** maxilla **d** maxilla, ventrolateral section. Scale bars: 20 µm.

**Figure 4.**
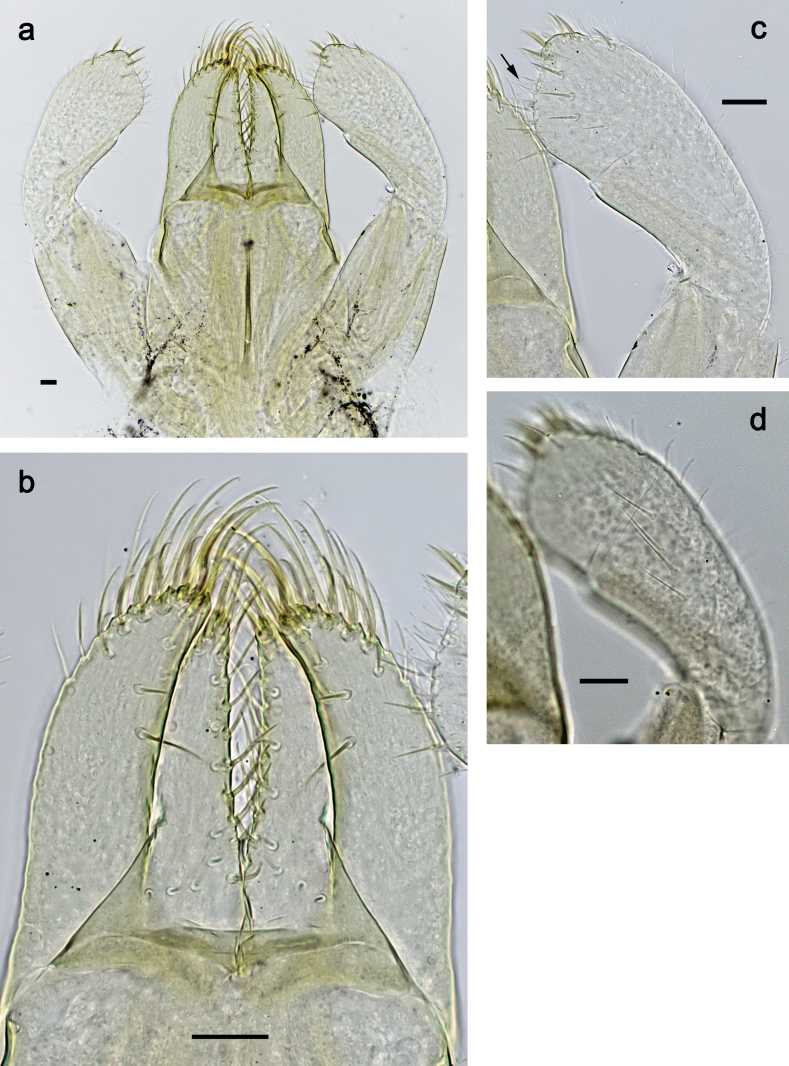
*Nigrobaetisplures* sp. nov., larva morphology **a** labium **b** glossae and paraglossae, ventral focus **c** labial palp, ventral focus (arrow: setae-like processes) **d** labial palp, dorsal focus. Scale bars: 20 µm.

**Figure 5.**
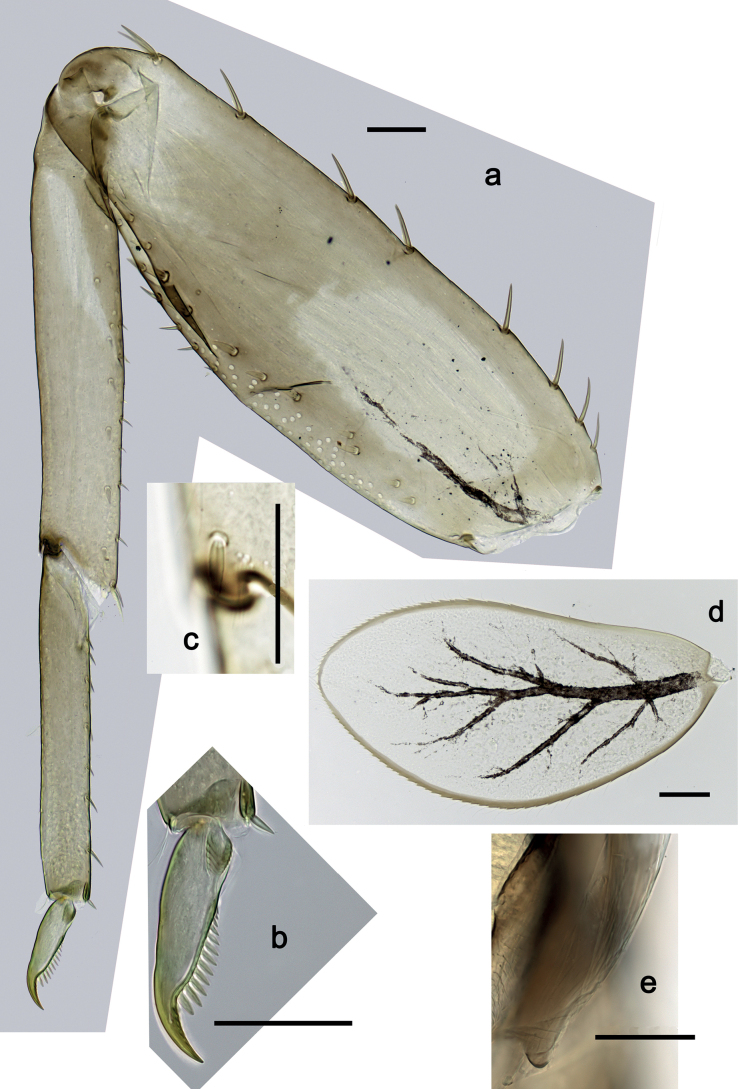
*Nigrobaetisplures* sp. nov., larva morphology **a** foreleg **b** fore claw **c** seta on dorsal apex of tibia **d** tergalius IV **e** right part of metanotum with hind protopteron. Scale bars: 50 µm.

**Figure 6.**
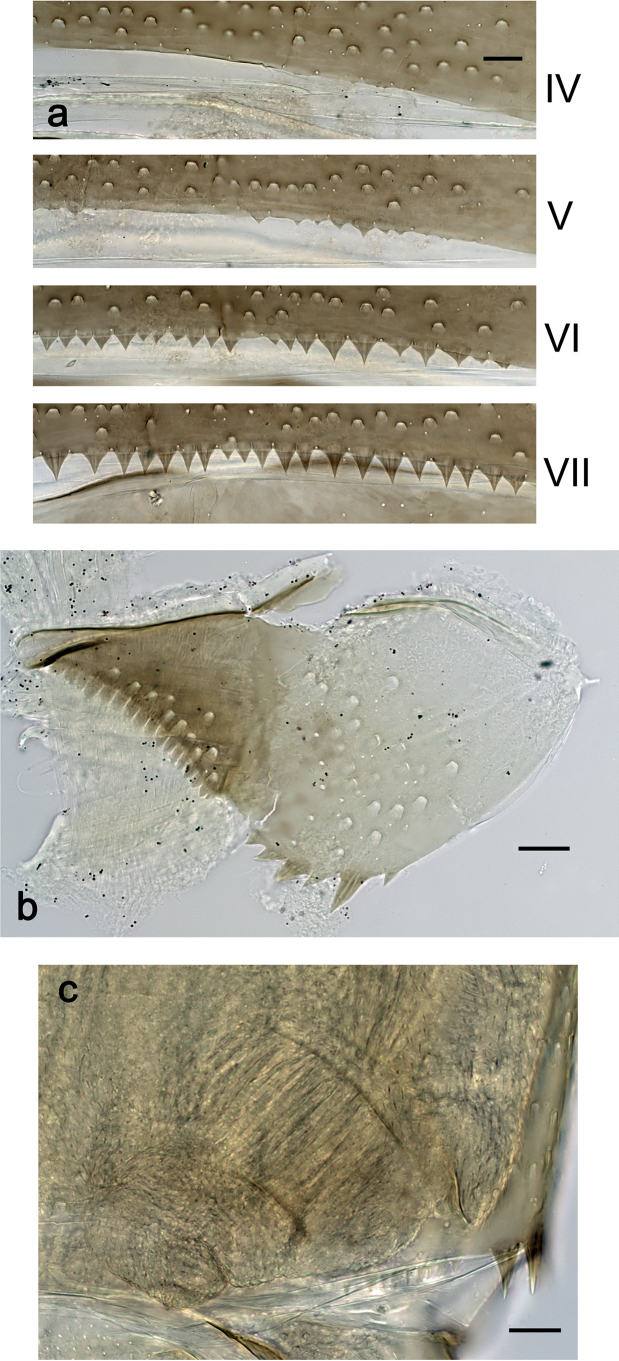
*Nigrobaetisplures* sp. nov., larva morphology **a** posterior margins of abdominal tergites IV–VII **b** paraproct **c** subimaginal gonostylus developing under cuticle of male last instar larva. Scale bars: 20 µm.

**Figure 7.**
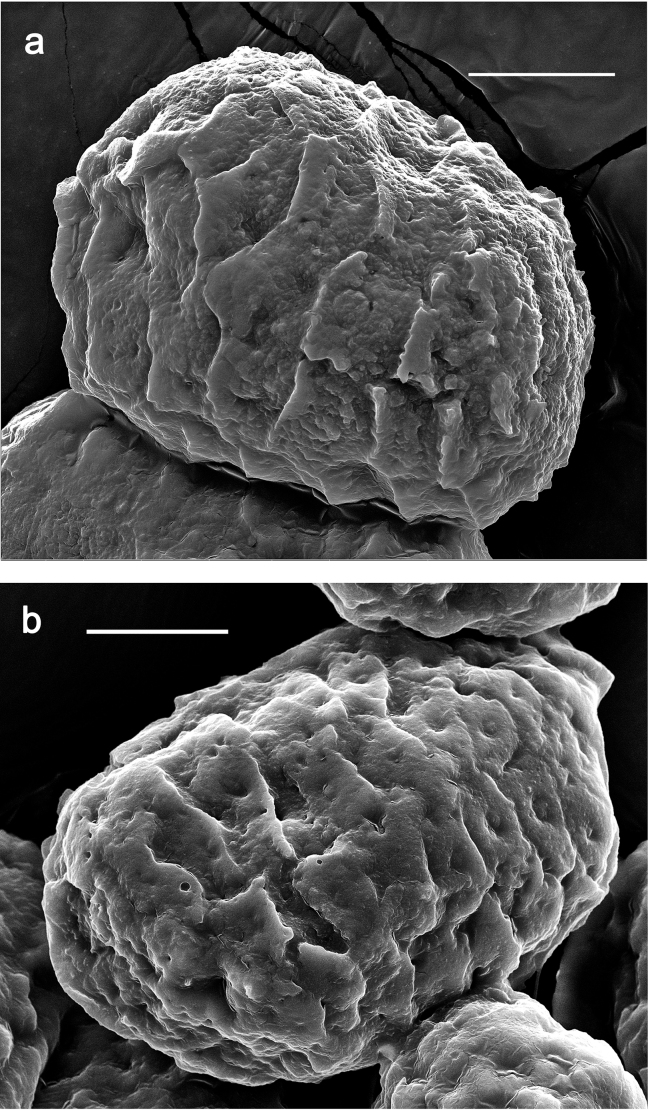
*Nigrobaetisplures* sp. nov., eggs. Scale bars: 20 µm.

**Larva** (Figs 1–7). Body length 3.3–4.9 mm. Cerci: ca.½ of body length. Paracercus: ca. ⅔ of cerci length. Antennae broken.

***Colouration*** (Fig. 1a–c). Head, thorax, and abdomen dorsally brown; abdominal terga VI and VII darker, abdominal terga VIII–X pale brown, except apex of tergum X brown. Head ventrally brown, thorax and abdomen ventrally pale brown, abdominal sterna VII and VIII darker. Legs pale brown, femur basomedially and distomedially with uncoloured areas. Caudalii brown.

***Labrum*** (Fig. 2a, b). Slightly conical, length 0.76× maximum width. Distal margin with medial emargination and a small process. Dorsally with medium, fine, simple setae scattered over surface; submedian seta and two long, simple, submarginal setae. Ventrally with marginal row of setae composed of anterolateral long, feathered setae and medial long, bifid, pectinate setae.

***Right mandible*** (Fig. 2c). Incisor and kinetodontium fused. Incisor with four denticles; kinetodontium with four denticles, inner margin of innermost denticle with row of thin setae. Prostheca stick-like, apicolaterally denticulate. Margin between prostheca and mola straight, with row of setae-like processes. Tuft of setae at apex of mola present.

***Left mandible*** (Fig. 2d). Incisor and kinetodontium fused. Incisor with four or five denticles; kinetodontium with three denticles. Prostheca robust, apically with small denticles and comb-shaped structure. Margin between prostheca and mola straight, with row of minute denticles. Tuft of setae at apex of mola absent.

***Hypopharynx and superlinguae*** (Fig. 3a). Lingua approx. as long as superlinguae. Lingua longer than broad; medial tuft of stout setae poorly developed, broad; distal half laterally not expanded. Superlinguae distally straight; lateral margins rounded; fine, long, simple setae along distal margin.

***Maxilla*** (Fig. 3b–d). Galea-lacinia ventrally with two simple, apical setae under canines. Medially with one spine-like seta and ca. four long, simple setae. Maxillary palp 1.3× as long as length of galea-lacinia; 2-segmented; palp segment II approx. as long as segment I; setae on maxillary palp fine, simple, scattered over surface of segments I and II; apex of last segment pointed.

***Labium*** (Fig. 4a–c). Glossa basally broad, narrowing toward apex; nearly as long as paraglossa; inner margin with ca. ten spine-like setae; apex with two long and one medium, robust setae; outer margin with ca. six spine-like setae; ventral surface with fine, simple, scattered setae. Paraglossa curved inward; apex rounded; with three rows of long, robust, distally pectinate setae in apical area and two medium, simple setae in inner anterolateral area; dorsally with row of three long, spine-like, simple setae near inner margin. Labial palp with segment I 1.2× length of segments II and III combined. Segment I ventrally with short, fine, simple setae. Segment II without protuberance; ventral surface with short, fine, simple setae; dorsally with row of four long, spine-like setae. Segment III sub-quadrangular; inner apical margin slightly concave, with some setae-like processes; apex with rounded protrusion; length subequal to width; ventrally with short, spine-like, simple setae and short, fine, simple setae.

***Hind protoptera*** (Fig. 5e) minute.

***Foreleg*** (Fig. 5a–c). Ratio of foreleg segments 1.5:1.0:0.8:0.3. ***Femur*.** Length ca. 3× maximum width. Dorsal margin with 7–11 curved, spine-like setae; length of setae 0.21× maximum width of femur. Apex rounded, with pair of spine-like setae. Stout, lanceolate setae scattered along ventral margin; femoral patch absent. ***Tibia*.** Dorsal margin with 0–3 short, spine-like setae, and with row of fine, simple setae; on apex one stout, apically rounded seta. Ventral margin with row of short, curved, spine-like setae, on apex some longer, spine-like, pectinate setae. Anterior surface with row of stout, lanceolate setae near ventral margin. Patellatibial suture present on basal half. ***Tarsus*.** Dorsal margin with row of fine, simple setae. Ventral margin with row of curved, spine-like setae. ***Claw*** with one row of 11 or 12 denticles; distally pointed; subapical setae absent.

***Middle and hind legs*.** As foreleg, but tibiae at dorsal margin with 4–6 spine-like setae.

***Abdominal terga*** (Fig. 6a). Surface with irregular rows of U-shaped scale bases. Posterior margin of terga: I–III smooth, without spines; IV and V with rudimentary spines or smooth; VI–IX with triangular, sharply pointed spines.

***Abdominal sterna*.** Posterior margins smooth, without spines.

***Tergalii*** (Fig. 5d). Present on segments I–VII. Margin with small denticles intercalating fine simple setae. Tracheae extending from main trunk to inner and outer margins. Tergalius I ca. ½ length of segment II; Tergalius IV as long as length of segments V and ½ VI combined; Tergalius VII as long as length of segments VIII and ½ IX combined.

***Paraproct*** (Fig. 6b). With 6–9 stout, marginal spines. Surface scattered with U-shaped scale bases. Cercotractor with numerous small, marginal spines.

***Subimaginal gonostyli*** (Fig. 6c) developing under cuticle of male last instar larvae folded in *Nigrobaetis*-type (Kluge 2004: fig. 29G).

**Adult stages.** Unknown.

**Eggs (Fig. 7a, b).** Ovoid; surface cone-like with numerous papillae-like structural elements (polygonal to rounded structure, centrally with a smaller, rounded, slightly elevated area surrounded by a round trench).

###### Etymology.

*Plures* in Latin meaning several, referring to the distribution of the species across several islands.

###### Distribution

**Figure 8.**
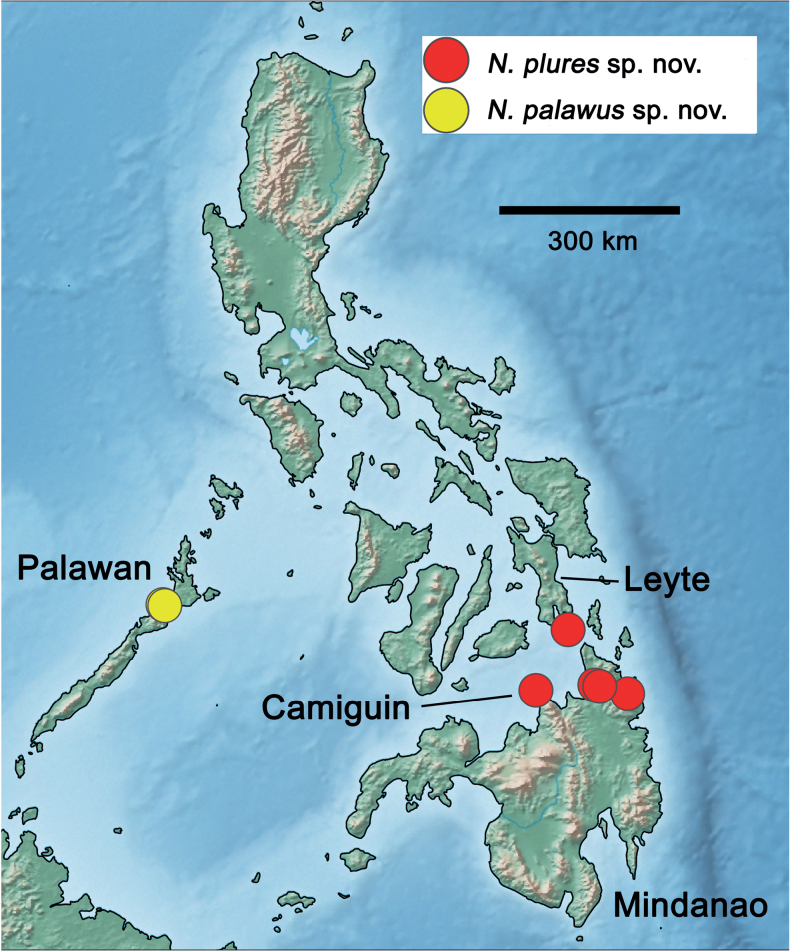
Distribution of *Nigrobaetis* species in the Philippines.

**(Fig. 8).** Philippines: Leyte, Mindanao, Camiguin.

###### Biological aspects.

The specimens were collected on altitudes of 30–660 m, mostly on bottom gravel in run or riffle, partly together with *Labiobaetiscamiguinensis* Kaltenbach, Garces & Gattolliat, 2020 (Camiguin) or *L.delocadoi* Kaltenbach, Garces & Gattolliat, 2020 (Leyte).

###### Type-material.

***Holotype*.** Philippines • larva; Leyte, Southern Leyte, Brgy. Malico, San Francisco, Taglibas River; 10°01'07"N, 125°12'35"E; 50 m; 19.-20. X. 2019; leg. Garces and Pelingen; on slide; GBIFCH00592356; PNM. ***Paratypes*.** Philippines • 4 larvae; same data as holotype; 3 on slides; GBIFCH00975650; AdMU; GBIFCH00515525, GBIFCH00975682; MZL; 1 in alcohol; GBIFCH00975649; AdMU • 1 larva; Mindanao, Surigao del Sur, Tandag, middle Tandag River; 09°03'33"N, 126°05'57"E; 30 m; 04.XII.2018; leg. Pangantihon; on slide; GBIFCH00592268; MZL • 1 larva; Mindanao, Agusan N, Cabadbaran, Del Pilar, Payas River; 09°11'34"N, 125°36'34"E; 660 m; 23. VI. 2018; leg. Pangantihon; on slide; GBIFCH00975656; AdMU • 2 larvae; Mindanao, Agusan N, Cabadbaran River; 09°10'15"N, 125°40'55"E; 240 m; 03. VI. 2018; leg. H. Freitag and Pangantihon; GBIFCH00975657, GBIFCH00975678; MZL • 6 larvae; Camiguin, Sagay, Bonbon, lower Binangawan River; 30 m, 09°06'39"N, 124°43'45"E; 30 m; 09.XII.2018; leg. H. Freitag; 4 on slides; GBIFCH00654914, GBIFCH00975652; MZL; GBIFCH00592248, GBIFCH00592249; AdMU; 2 in alcohol; GBIFCH00975651, GBIFCH00975653; AdMU.

##### ﻿ Nigrobaetis palawus sp. nov.

Taxon classificationAnimaliaEphemeropteraBaetidae

E6FF716D-073B-5FDB-8961-6E4EEC6C97BA

https://zoobank.org/26E833A0-1208-4F53-9FF2-EAE3A9F15E29

**Figure 9.**
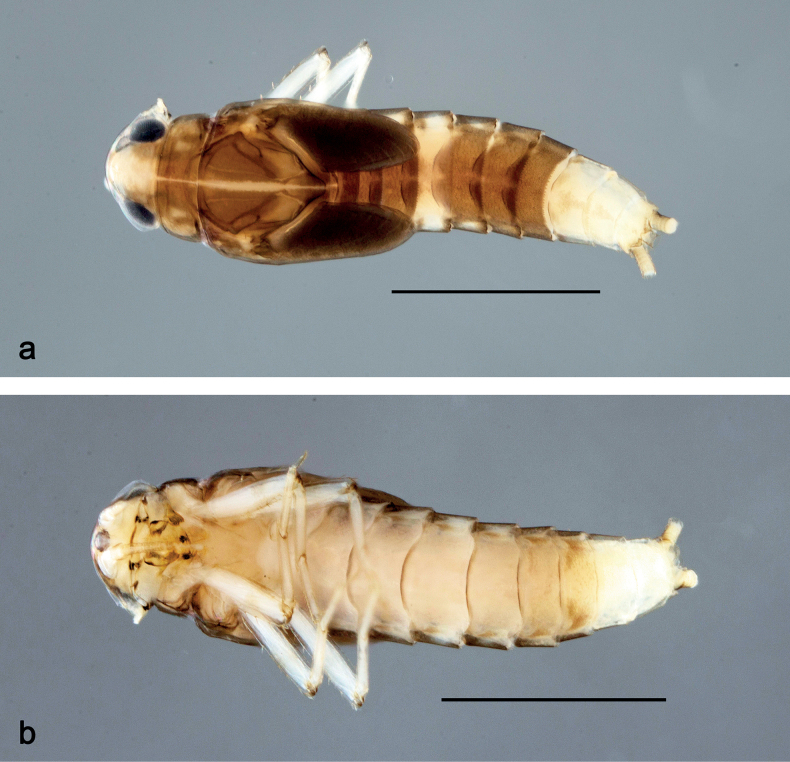
*Nigrobaetispalawus* sp. nov., larva habitus **a** dorsal view **b** ventral view. Scale bars: 1 mm.

**Figure 10.**
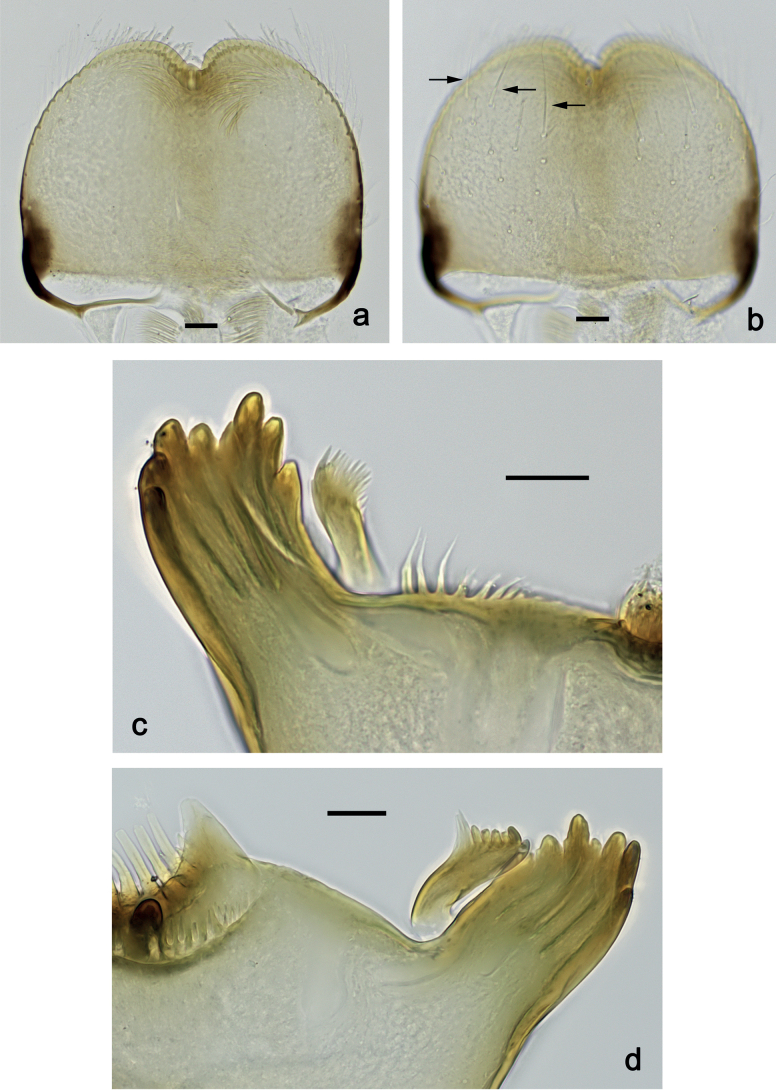
*Nigrobaetispalawus* sp. nov., larva morphology **a** labrum, ventral focus **b** labrum, dorsal focus (arrows: submedian and submarginal setae) **c** right mandible **d** left mandible. Scale bars: 20 µm.

**Figure 11.**
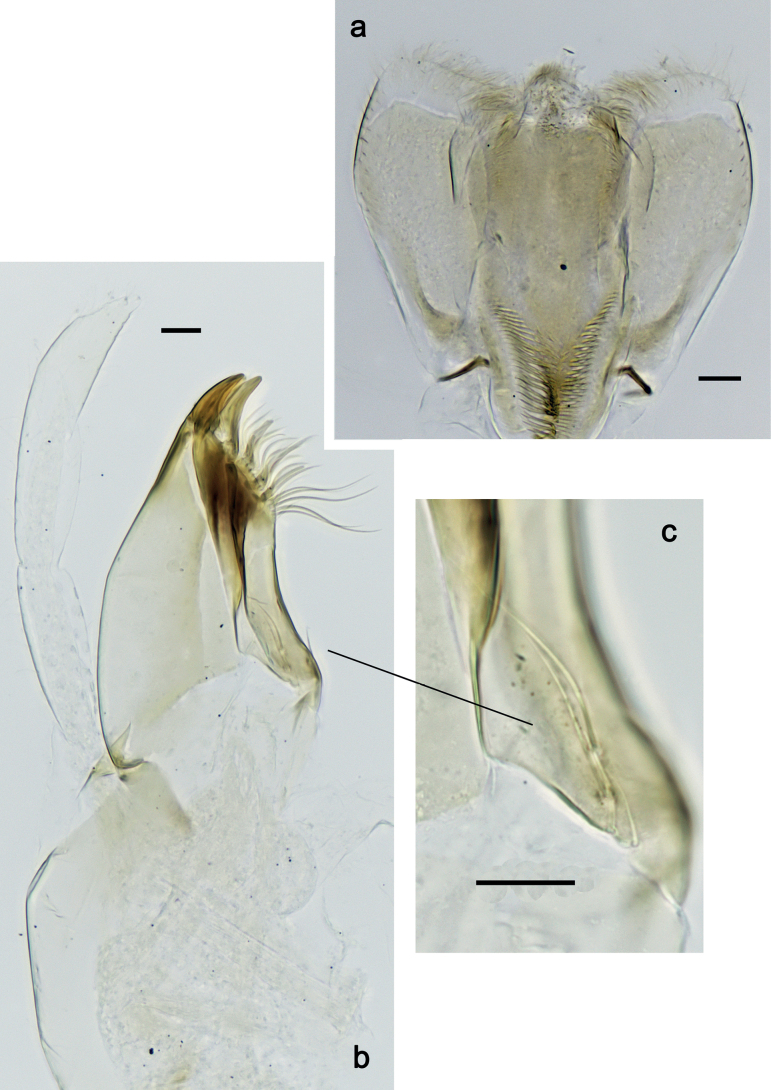
*Nigrobaetispalawus* sp. nov., larva morphology **a** hypopharynx and superlinguae**b** maxilla **c** maxilla, ventrolateral section. Scale bars: 20 µm.

**Figure 12.**
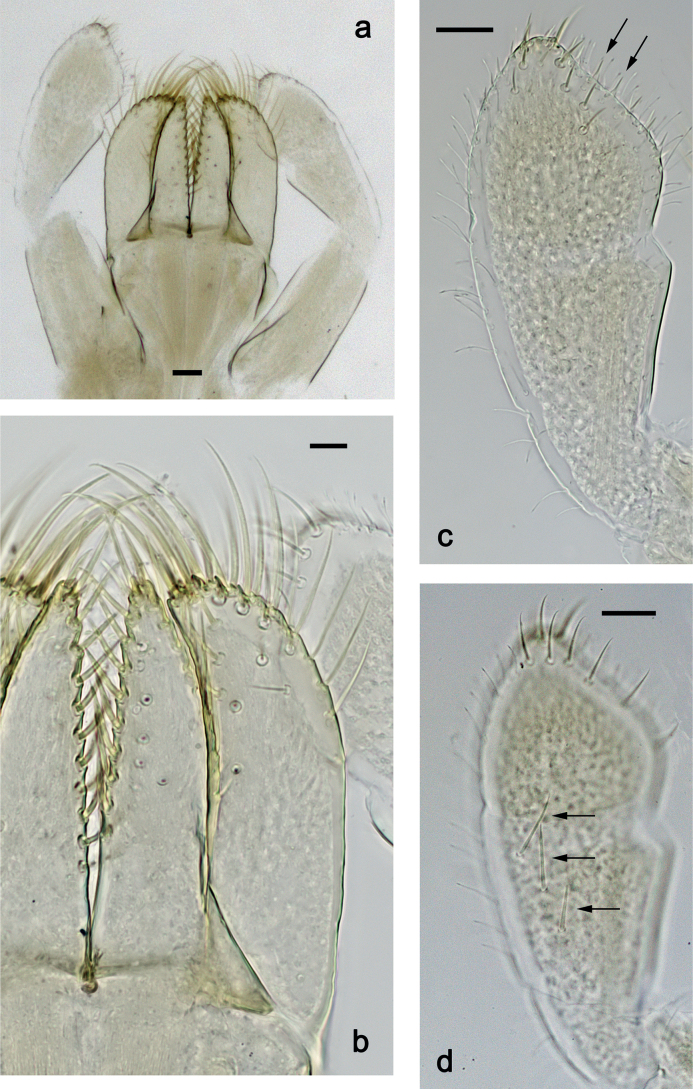
*Nigrobaetispalawus* sp. nov., larva morphology **a** labium **b** glossae and paraglossae, ventral focus **c** labial palp, ventral focus (arrows: setae-like processes) **d** labial palp, dorsal focus. Scale bars: 20 µm.

**Figure 13.**
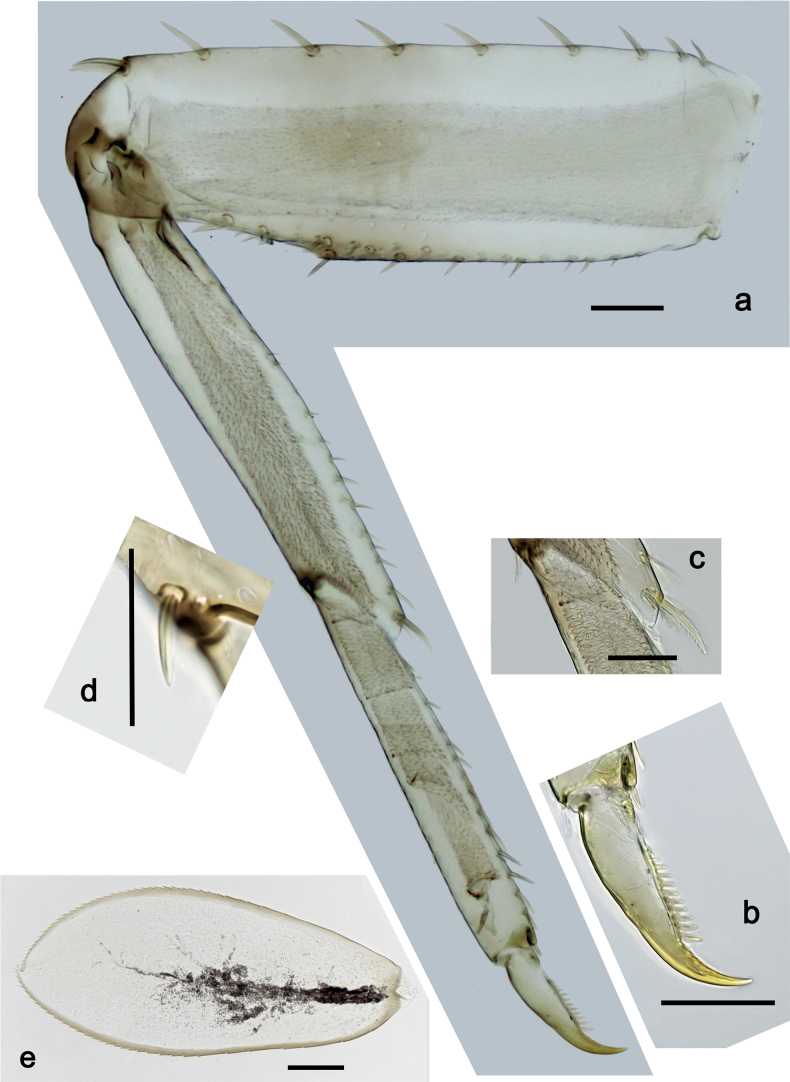
*Nigrobaetispalawus* sp. nov., larva morphology **a** foreleg **b** fore claw **c** ventral apex of tibia **d** dorsal apex of tibia **e** tergalius VI. Scale bars: 50 µm.

**Figure 14.**
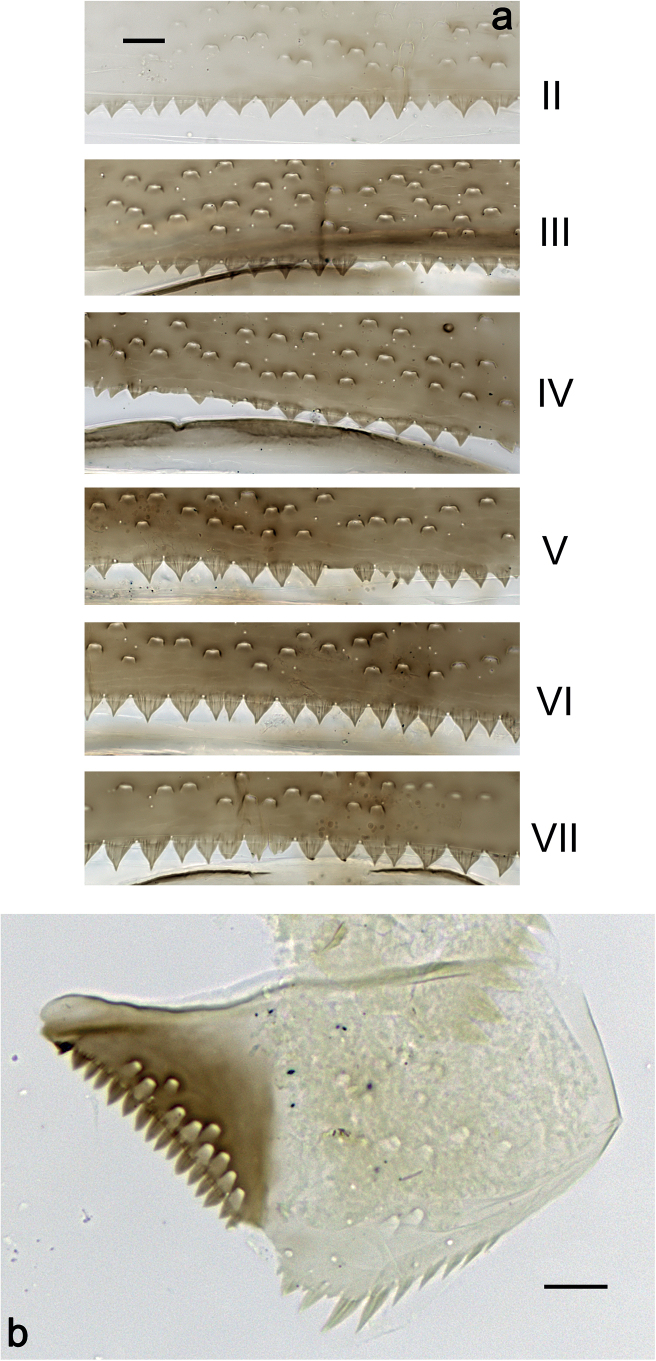
*Nigrobaetispalawus* sp. nov., larva morphology **a** posterior margins of abdominal tergites II–VII **b** paraproct. Scale bars: 20 µm.

Figs 8 , 9 , 10 , 11 , 12 , 13 , 14

###### Differential diagnosis.

**Larva.** Following combination of characters: A) dorsal surface of labrum with submedian seta and two long, simple setae in submarginal position (Fig. 10b); B) right mandible: incisor with four denticles, kinetodontium with four denticles (Fig. 10c); C) left mandible: incisor with four or five denticles, kinetodontium with three denticles; margin between prostheca and mola smooth, without denticles (Fig. 10d); D) fore femur length ca. 3× maximum width, dorsal margin with 8–14 curved, spine-like setae (Fig. 13a); E) claw with 11 or 12 denticles (Fig. 13b); F) hind protoptera absent; G) tergalii on abdominal segments I–VII; H) paraproct with ca. 12 marginal spines (Fig. 14b); I) posterior margins of abdominal terga: I smooth, without spines; II–IX with triangular spines (Fig. 14a).

###### Description.

**Larva** (Figs 9–14). Body length 2.8–3.9 mm. Caudalii broken. Antennae broken.

***Colouration*** (Fig. 9a, b). Head dorsally pale brown, basolaterally darker; thorax and abdomen dorsally brown; abdominal terga IV and VIII–X pale brown. Head, thorax, and abdomen ventrally pale brown, abdominal sterna VIII–X brighter. Legs pale brown, femur apically brown, with distomedial spot. Caudalii pale brown.

***Labrum*** (Fig. 10a, b). Length 0.7× maximum width. Distal margin with medial emargination and a small process. Dorsally with medium, fine, simple setae scattered over surface; submedian seta and two long, simple, submarginal setae. Ventrally with marginal row of setae composed of anterolateral long, feathered setae and medial long, bifid, pectinate setae.

***Right mandible*** (Fig. 10c). Incisor and kinetodontium fused. Incisor with four denticles; kinetodontium with four denticles, inner margin of innermost denticle with row of thin setae. Prostheca stick-like, apicolaterally denticulate. Margin between prostheca and mola straight, with row of setae-like processes. Tuft of setae at apex of mola present.

***Left mandible*** (Fig. 10d). Incisor and kinetodontium fused. Incisor with four denticles; kinetodontium with three denticles. Prostheca robust, apically with small denticles and comb-shaped structure. Margin between prostheca and mola straight, smooth without denticles. Tuft of setae at apex of mola absent.

***Hypopharynx and superlinguae*** (Fig. 11a). Lingua approx. as long as superlinguae. Lingua longer than broad; medial tuft of stout setae poorly developed, broad; distal half laterally not expanded. Superlinguae distally straight; lateral margins rounded; fine, long, simple setae along distal margin.

***Maxilla*** (Fig. 11b, c). Galea-lacinia ventrally with two simple, apical setae under canines. Medially with one spine-like seta and ca. four long, simple setae. Maxillary palp approx. as long as length of galea-lacinia; 2-segmented; palp segment II approx. 1.1× as long as segment I; setae on maxillary palp fine, simple, scattered over surface of segments I and II; apex of last segment pointed.

***Labium*** (Fig. 12a–d). Glossa basally broad, narrowing toward apex; as long as paraglossa; inner margin with ca. ten spine-like setae; apex with two long and one medium, robust setae; outer margin with ca. four spine-like setae; ventral surface with fine, simple, scattered setae. Paraglossa curved inward; apex rounded; with three rows of long, robust, distally pectinate setae in apical area and two medium, simple setae in anteromedial area; dorsally with row of three long, spine-like, simple setae near inner margin. Labial palp with segment I 0.9× length of segments II and III combined. Segment I ventrally with short, fine, simple setae. Segment II without protuberance; ventral surface with short, fine, simple setae; dorsally with row of three or four long, spine-like setae. Segment III pentagonal; inner apical margin with some setae-like processes; length 1.1× width; ventrally with short, spine-like, simple setae and short, fine, simple setae.

***Hind protoptera***: Absent.

***Foreleg*** (Fig. 13a–d). Ratio of foreleg segments 1.3:1.0:0.8:0.3. ***Femur*.** Length ca. 3× maximum width. Dorsal margin with 8–14 curved, spine-like setae; length of setae 0.27× maximum width of femur. Apex rounded, with pair of spine-like setae. Stout, lanceolate setae scattered along ventral margin; femoral patch absent. ***Tibia*.** Dorsal margin without or with one short, spine-like seta; on apex one stout, apically rounded seta. Ventral margin with row of short, curved, spine-like setae, on apex two longer, spine-like, pectinate setae. Anterior surface with some stout, lanceolate setae near ventral margin. Patellatibial suture present on basal half area. ***Tarsus*.** Dorsal margin almost bare. Ventral margin with row of curved, spine-like setae. ***Claw*** with one row of 11 or 12 denticles; distally pointed; subapical setae absent.

***Middle and hind legs*.** As foreleg, but *tibia* at dorsal margin with 1–4 spine-like setae.

***Abdominal terga*** (Fig. 14a). Surface with irregular rows of U-shaped scale bases. Posterior margin of terga: I smooth, without spines; II–IX with triangular, pointed spines.

***Abdominal sterna*.** Posterior margins smooth, without spines.

***Tergalii*** (Fig. 13e). Present on segments I–VII. Margin with small denticles intercalating fine simple setae. Tracheae extending from main trunk to inner and outer margins. Tergalius II as long as length of segments III and ½ IV combined; Tergalius VII as long as length of segments VIII and ½ IX combined.

***Paraproct*** (Fig. 14b). With 10–12 stout, marginal spines. Surface scattered with U-shaped scale bases. Cercotractor with numerous small, marginal spines.

**Adult stages.** Unknown.

###### Etymology.

Referring to the island of Palawan, where the species was collected.

###### Distribution

**(Fig. 8).** Philippines: Palawan.

###### Biological aspects.

The specimens were collected below 100 m in primary and secondary forest, on bottom gravel and on root packs in the run.

###### Type-material.

***Holotype*.** Philippines • larva; Palawan, San Vincente, waterfall 4km NE Port Barton; 10°23'35"N, 119°09'27"E; 30 m; 18.vi.1997; leg. J. Mendoza; on slide; GBIFCH00975661; PNM. ***Paratypes*.** Philippines • 3 larvae; same data as holotype; 3 on slides; GBIFCH00592651; AdMU; GBIFCH0092646, GBIFCH00592647; MZL • 1 larva; Palawan, Roxas, Bgy. Port Barton, mountain river; 10°22'40"N, 119°11'05"E; 90 m; 20.vi.1997; leg. J. Mendoza; in alcohol; GBIFCH00975662; AdMU.

##### ﻿ Nigrobaetis sumbensis sp. nov.

Taxon classificationAnimaliaEphemeropteraBaetidae

D86B5CC0-B376-5093-8BA1-14203937E3EC

https://zoobank.org/C3E8FA2E-3517-46D9-A617-539AD9FB49D2

**Figure 15.**
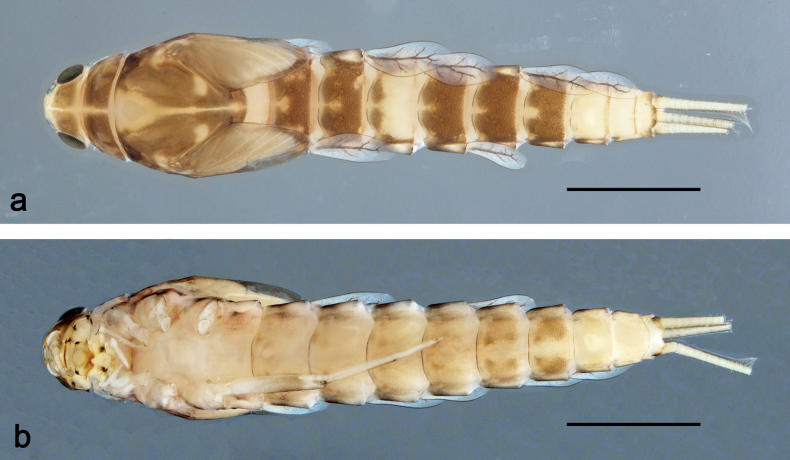
*Nigrobaetissumbensis* sp. nov., larva habitus **a** dorsal view **b** ventral view. Scale bars: 1 mm.

**Figure 16.**
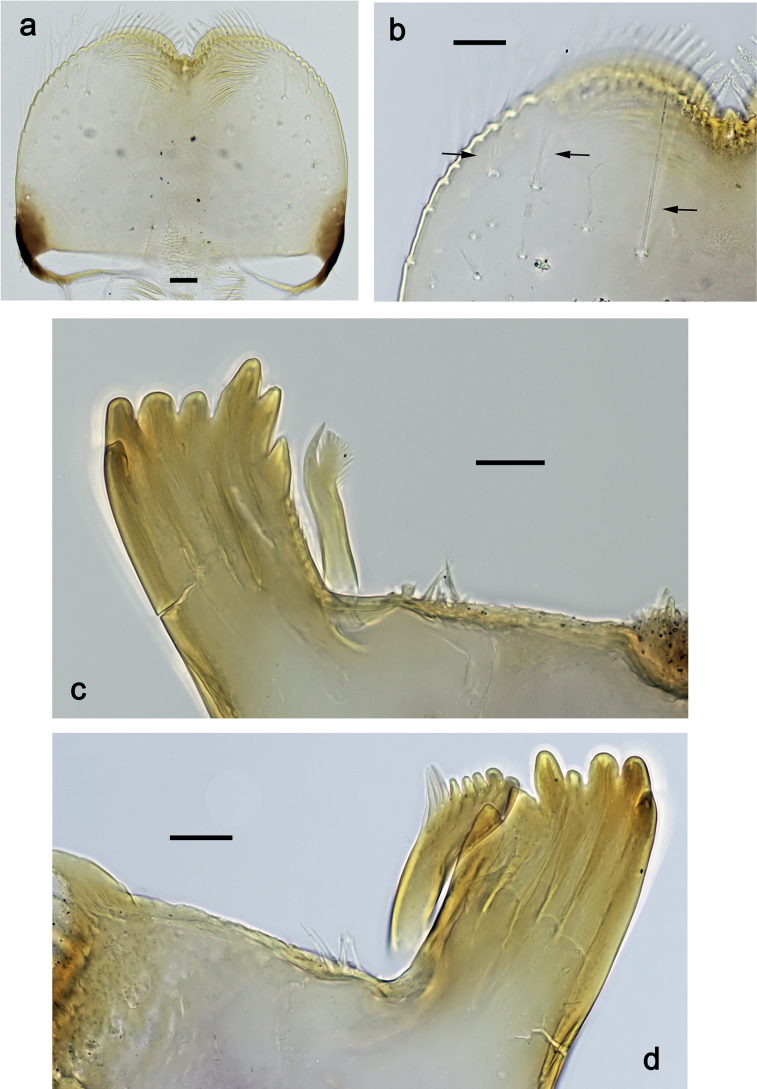
*Nigrobaetissumbensis* sp. nov., larva morphology **a** labrum, ventral focus **b** labrum, dorsal focus (arrows: submedian and submarginal setae) **c** right mandible **d** left mandible. Scale bars: 20 µm.

**Figure 17.**
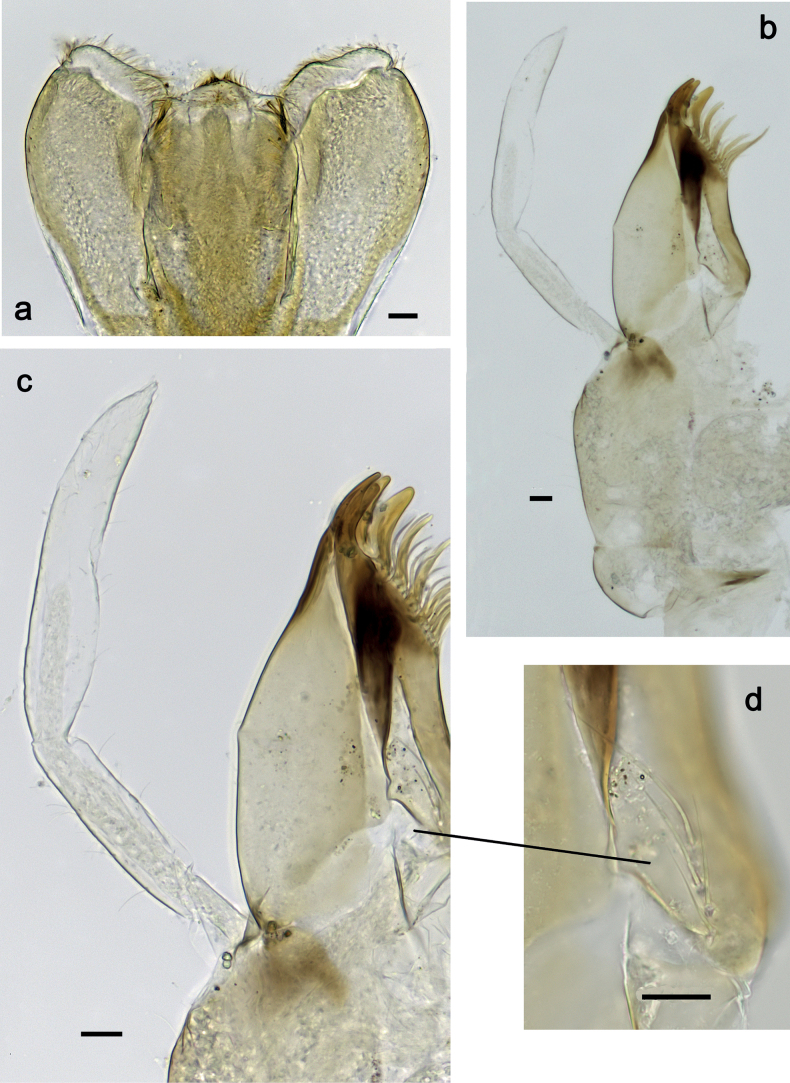
*Nigrobaetissumbensis* sp. nov., larva morphology **a** hypopharynx and superlinguae**b, c** maxilla **d** maxilla, ventrolateral section. Scale bars: 20 µm.

**Figure 18.**
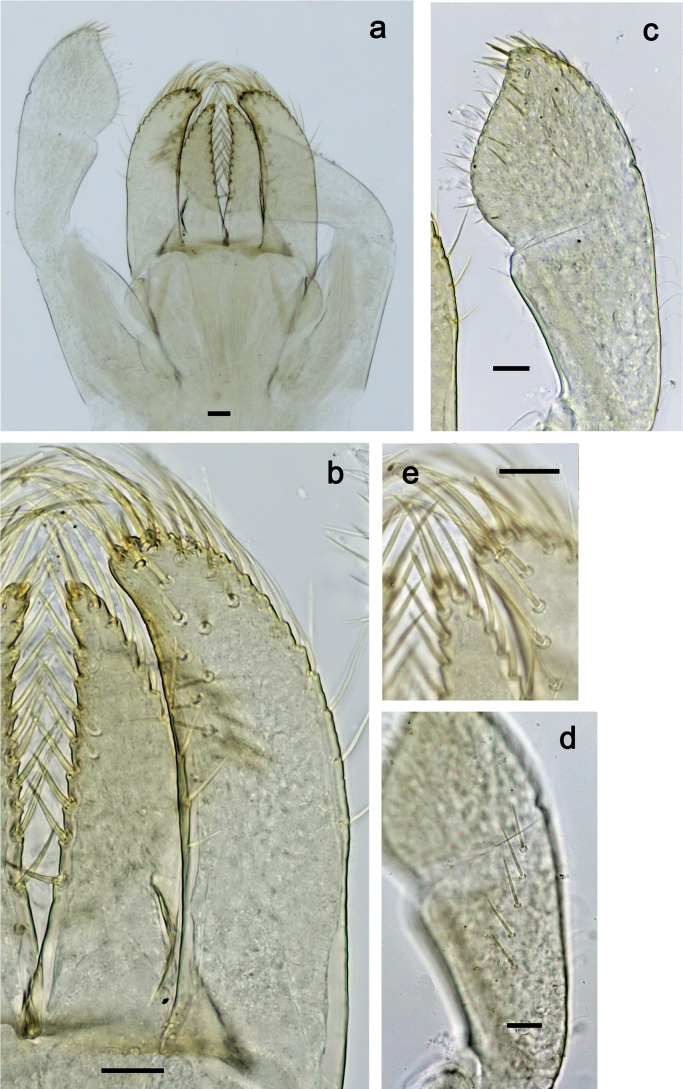
*Nigrobaetissumbensis* sp. nov., larva morphology **a** labium **b** glossae and paraglossae, ventral focus **c** labial palp, ventral focus **d** labial palp, dorsal focus **e** apex of glossa and paraglossa, dorsal focus. Scale bars: 20 µm.

**Figure 19.**
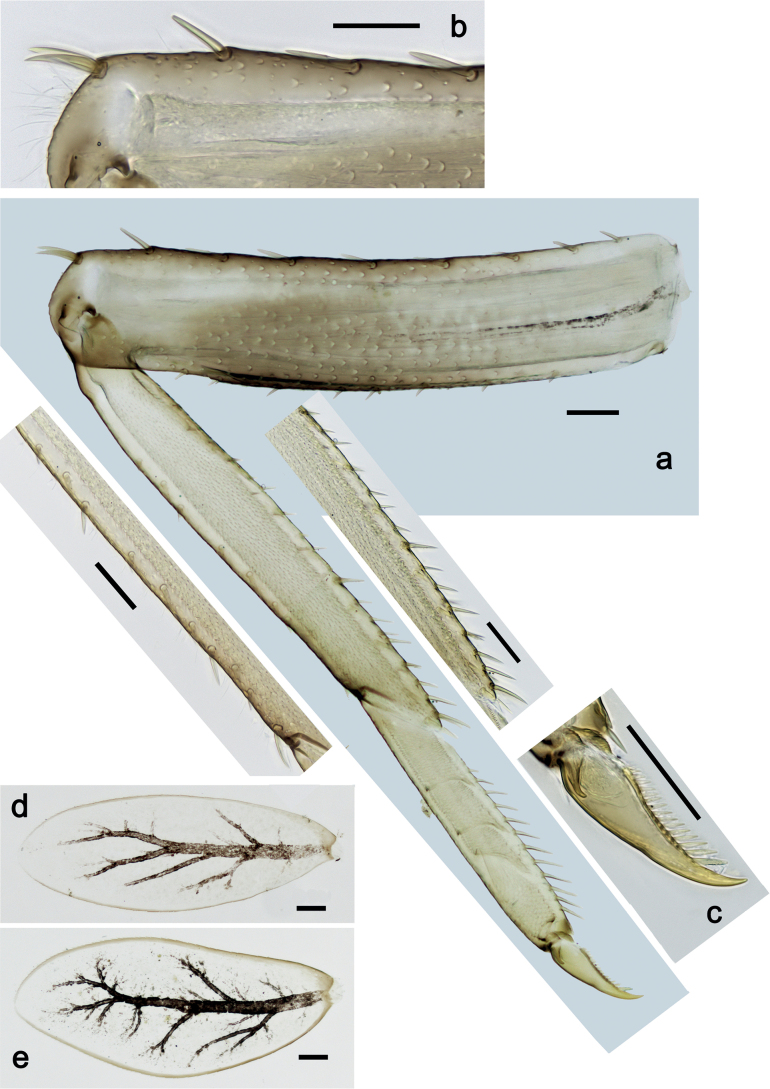
*Nigrobaetissumbensis* sp. nov., larva morphology **a** foreleg **b** fore femur apex **c** fore claw **d** tergalius I **e** tergalius IV. Scale bars: 50 µm.

**Figure 20.**
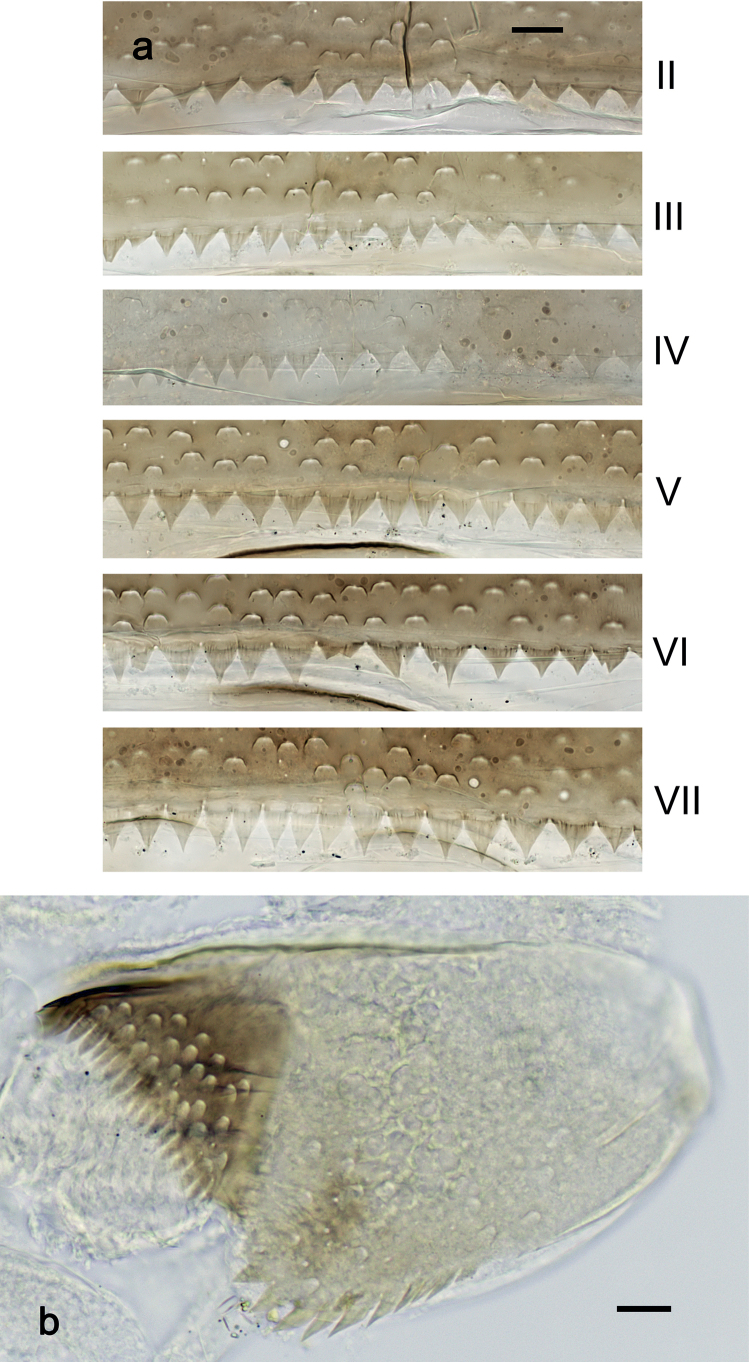
*Nigrobaetissumbensis* sp. nov., larva morphology **a** posterior margins of abdominal tergites II–VII **b** paraproct. Scale bars: 20 µm.

Figs 15 , 16 , 17 , 18 , 19 , 20 , 40c

###### Differential diagnosis.

**Larva.** Following combination of characters: A) dorsal surface of labrum with submedian seta and two long, simple setae in submarginal position (Fig. 16b); B) right mandible: incisor with four denticles, kinetodontium with three denticles (Fig. 16c); C) left mandible: incisor with four denticles, kinetodontium with three denticles; margin between prostheca and mola straight, with four or five long, setae-like processes (Fig. 16d); D) fore femur very slender, length ca. 4.8× maximum width, dorsal margin with eight or nine curved, spine-like setae (Fig. 19a); E) tibia dorsally with row of spine-like setae; F) claw with 14–17 relatively long denticles (Fig. 19c); G) hind protoptera absent; H) tergalii on abdominal segments I–VII; I) paraproct with 10–14 marginal spines (Fig. 20b); J) posterior margins of abdominal terga: I smooth, without spines; II–IX with triangular, pointed spines (Fig. 20a).

###### Description.

**Larva** (Figs 15–20). Body length 3.7–4.6 mm. Caudalii broken. Antennae broken.

***Colouration*** (Fig. 15a, b). Head, thorax, and abdomen dorsally brown, with lively bright pattern as in Fig. 15a. Noticeable are the bright beige abdominal terga I, IV (with brown marks), and VIII–X. Head, thorax, and abdomen ventrally pale brown, abdominal sterna VIII–X brighter. Legs pale brown, femur darker in distal 2/3. Caudalii bright beige.

***Labrum*** (Fig. 16a, b). Length 0.7× maximum width. Distal margin with medial emargination and a small process. Dorsally with medium, fine, simple setae scattered over surface; submedian seta and two long, simple, submarginal setae. Ventrally with marginal row of setae composed of lateral and anterolateral long, feathered setae and medial long, bifid, pectinate setae.

***Right mandible*** (Fig. 16c). Incisor and kinetodontium fused. Incisor with four denticles; kinetodontium with three denticles, inner margin of innermost denticle without row of thin setae. Prostheca stick-like, apicolaterally denticulate. Margin between prostheca and mola straight, with row of setae-like processes. Tuft of setae at apex of mola present.

***Left mandible*** (Fig. 16d). Incisor and kinetodontium fused. Incisor with four denticles; kinetodontium with three denticles. Prostheca robust, apically with small denticles and comb-shaped structure. Margin between prostheca and mola straight, with four or five long, setae-like processes. Tuft of setae at apex of mola absent.

***Hypopharynx and superlinguae*** (Fig. 17a). Lingua shorter than superlinguae. Lingua longer than broad; medial tuft of stout setae poorly developed, broad; distal half laterally not expanded. Superlinguae distally straight; lateral margins rounded; fine, long, simple setae along distal margin.

***Maxilla*** (Fig. 17b–d). Galea-lacinia ventrally with two simple, apical setae under canines. Medially with one spine-like seta and ca. four long, simple setae. Maxillary palp ca. 1.3× as long as length of galea-lacinia; 2-segmented; palp segment II ca. 1.3× as long as segment I; setae on maxillary palp fine, simple, scattered over surface of segments I and II; apex of last segment pointed.

***Labium*** (Fig. 12a–e). Glossa basally broad, narrowing toward apex; slightly shorter than paraglossa; inner margin with nine or ten spine-like setae; apex with two long and one medium, robust setae; outer margin with ca. eight spine-like setae; ventral surface with fine, simple, scattered setae. Paraglossa curved inward; apex rounded; with three rows of long, robust, distally pectinate setae in apical area and ca. four medium, simple setae in anteromedial area; dorsally with row of four long, spine-like, simple setae near inner margin. Labial palp with segment I 0.7× length of segments II and III combined. Segment I ventrally with short, fine, simple setae. Segment II without protuberance; ventral surface with short, fine, simple setae; dorsally with row of ca. five long, spine-like setae. Segment III slightly pentagonal, inner distal margin slightly concave, with few setae-like processes; length ca. 1.2× maximum width; ventrally with short, spine-like, simple setae and short, fine, simple setae.

***Hind protoptera***: Absent.

***Foreleg*** (Fig. 19a–c). Ratio of foreleg segments 1.3:1.0:0.6:0.2. ***Femur*.** Very slender, length ca. 4.8× maximum width. Dorsal margin slightly concave, with eight or nine curved, spine-like setae; length of setae 0.29× maximum width of femur. Apex rounded, with pair of spine-like setae and medium, fine, simple setae. Row of stout, lanceolate setae on ventral margin; femoral patch absent. ***Tibia*.** Dorsal margin with row of medium, spine-like setae, on apex one seta somewhat longer and with rounded apex. Ventral margin with row of short to medium curved, spine-like setae, on apex two longer, spine-like, pectinate setae. Anterior surface with stout, lanceolate setae along ventral margin. Patellatibial suture present on basal half. ***Tarsus*.** Dorsal margin bare. Ventral margin with row of curved, spine-like setae. ***Claw*** with one row of 14–17 relatively long denticles; distally pointed; subapical setae absent.

***Middle and hind legs*.** As foreleg.

***Abdominal terga*** (Fig. 20a). Surface with irregular rows of U-shaped scale bases. Posterior margin of terga: I smooth, without spines; II–IX with triangular, pointed spines.

***Abdominal sterna*.** Posterior margin of sterna: I–V smooth, without spines; VI with rudimentary spines; VII–IX with triangular spines.

***Tergalii*** (Fig. 19d, e). Present on segments I–VII. Margin with small denticles intercalating fine simple setae. Tracheae extending from main trunk to inner and outer margins. Tergalius I as long as length of segments II and ⅓ III combined; Tergalius IV as long as length of segments V and VI combined; Tergalius VII reaching beginning of segment X.

***Paraproct*** (Fig. 20b). With 10–14 stout, marginal spines. Surface scattered with U-shaped scale bases. Cercotractor with numerous small, marginal spines.

**Adult stages.** Unknown.

###### Etymology.

Referring to the island of Sumba, where the species was collected.

###### Distribution

**(Fig. 40c).** Indonesia, Sumba.

###### Biological aspects.

The specimens were collected at an altitude of 400 m.

###### Type-material.

***Holotype*.** Indonesia • larva; Sumba, Waikelo. Stream; 09°35'45"S, 119°20'25"E; 400 m; 27.ix.2011; leg. M. Balke; (SUA04); on slide; GBIFCH00592616; MZB. ***Paratypes*.** Indonesia • 2 larvae; same data as holotype; 2 on slides; GBIFCH00592660, GBIFCH00975677; MZL.

##### ﻿ Nigrobaetis suma sp. nov.

Taxon classificationAnimaliaEphemeropteraBaetidae

1B7FAD5F-BE89-5545-9B52-5ABDCC6C537D

https://zoobank.org/BD78CF0A-B875-4035-BA4E-4F615DB810CE

Figs 21 , 22 , 23 , 24 , 25 , 26 , 27 , 40c

###### Differential diagnosis.

**Larva.** Following combination of characters: A) dorsal surface of labrum with submedian seta and two long, simple setae in submarginal position (Fig. 22b); B) right mandible: incisor with four denticles, kinetodontium with four denticles (Fig. 22c); C) left mandible: incisor with four denticles, kinetodontium with three denticles; margin between prostheca and mola straight, with row of short, setae-like processes (Fig. 22d); D) fore femur length ca. 3× maximum width, dorsal margin with ca. 11 curved, spine-like setae (Fig. 25a); E) tibia dorsally bare, distally with one stout, apically rounded seta (Fig. 25a, d); F) claw with 11 or 12 denticles (Fig. 25c); G) hind protoptera well developed (Fig. 25g); H) tergalii on abdominal segments I–VII; I) paraproct with six or seven marginal spines (Fig. 26b); J) posterior margins of abdominal terga: I–IV(V) smooth, without spines; (V–)VI with rudimentary spines; VII–IX with triangular, pointed spines (Fig. 26a).

###### Description.

**Figure 21.**
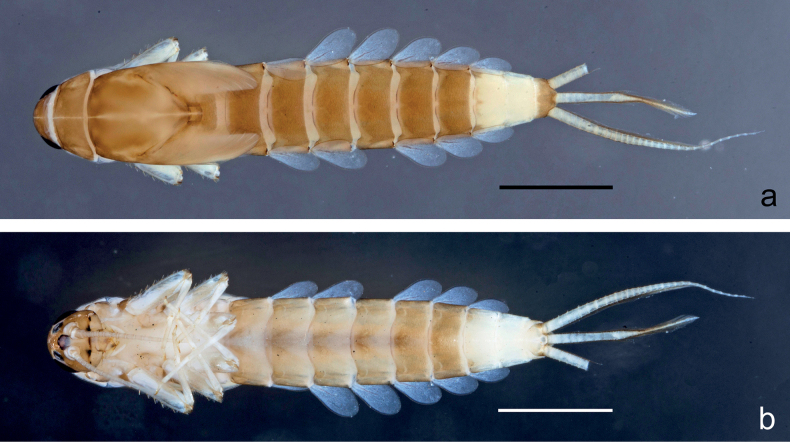
*Nigrobaetissuma* sp. nov., larva habitus **a** dorsal view **b** ventral view. Scale bars: 1 mm.

**Figure 22.**
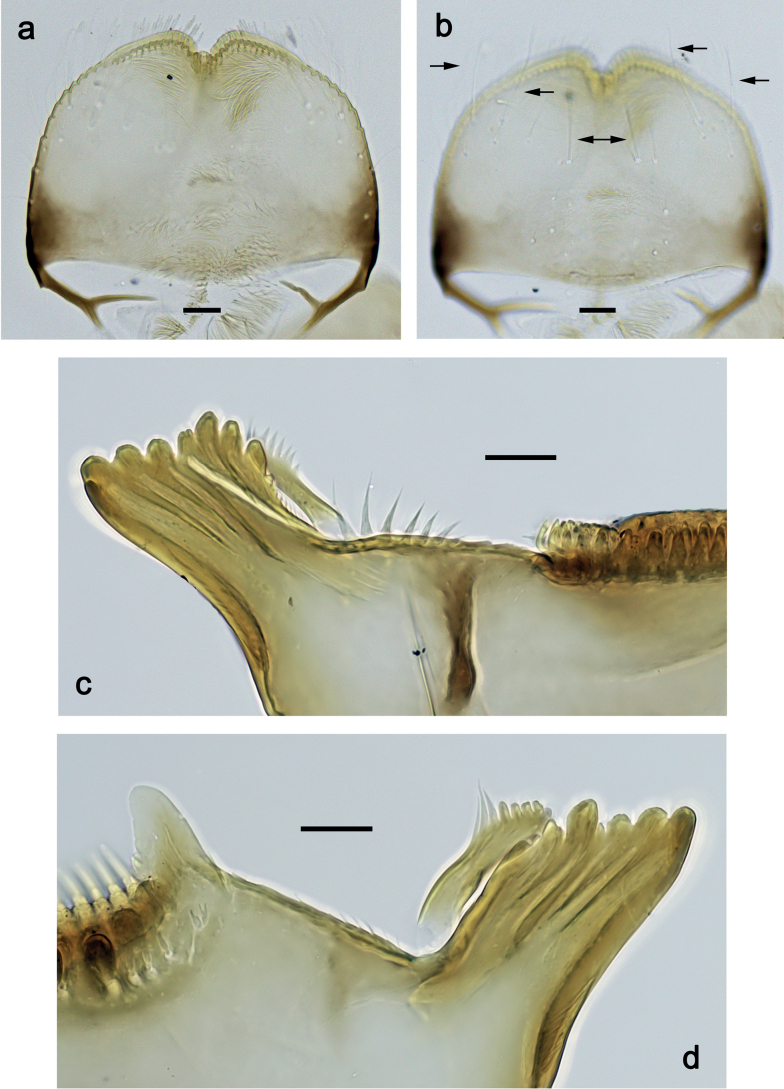
*Nigrobaetissuma* sp. nov., larva morphology **a** labrum, ventral focus **b** labrum, dorsal focus (arrows: submedian and submarginal setae) **c** right mandible **d** left mandible. Scale bars: 20 µm.

**Figure 23.**
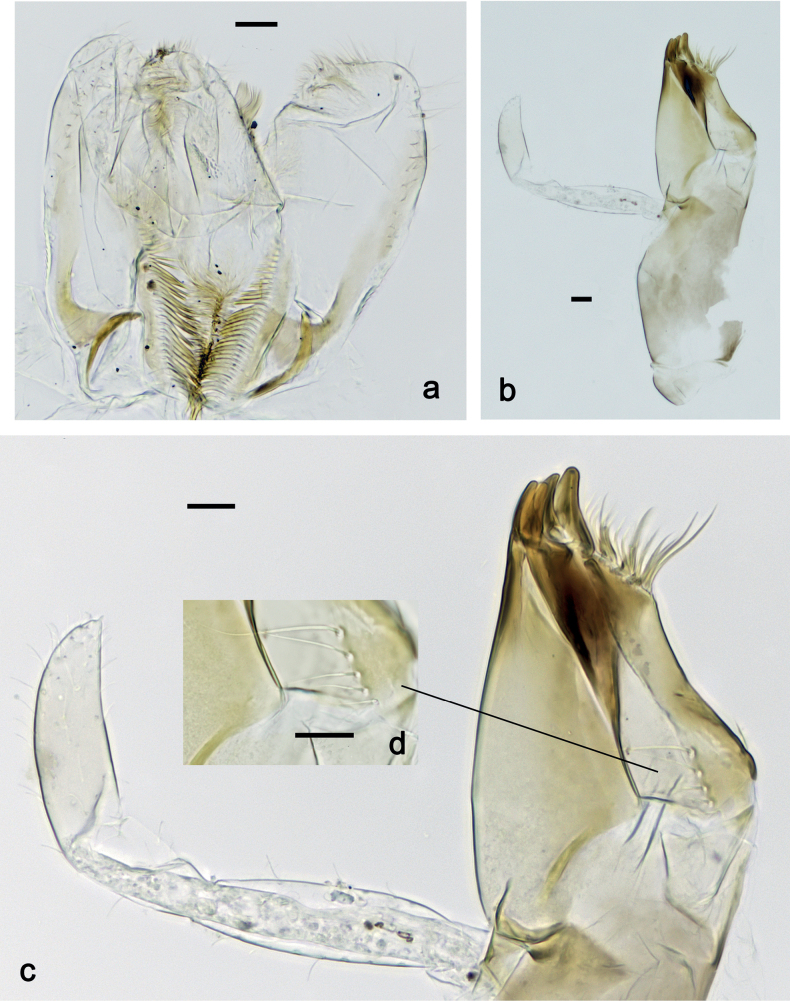
*Nigrobaetissuma* sp. nov., larva morphology **a** hypopharynx and superlinguae**b, c** maxilla **d** maxilla, ventrolateral section. Scale bars: 20 µm.

**Figure 24.**
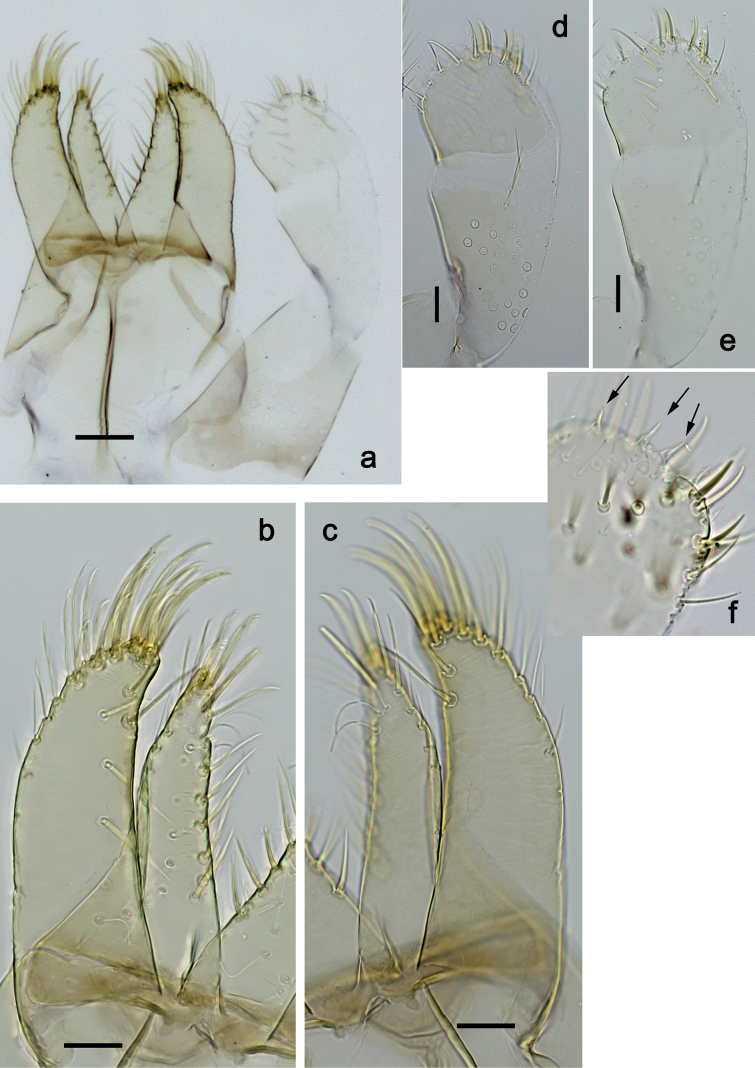
*Nigrobaetissuma* sp. nov., larva morphology **a** labium **b** glossa and paraglossa, ventral focus **c** glossa and paraglossa, dorsal focus **d** labial palp, dorsal focus **e** labial palp, ventral focus **f** labial palp, inner apical margin of segment III (arrows: setae-like processes). Scale bars: 20 µm.

**Figure 25.**
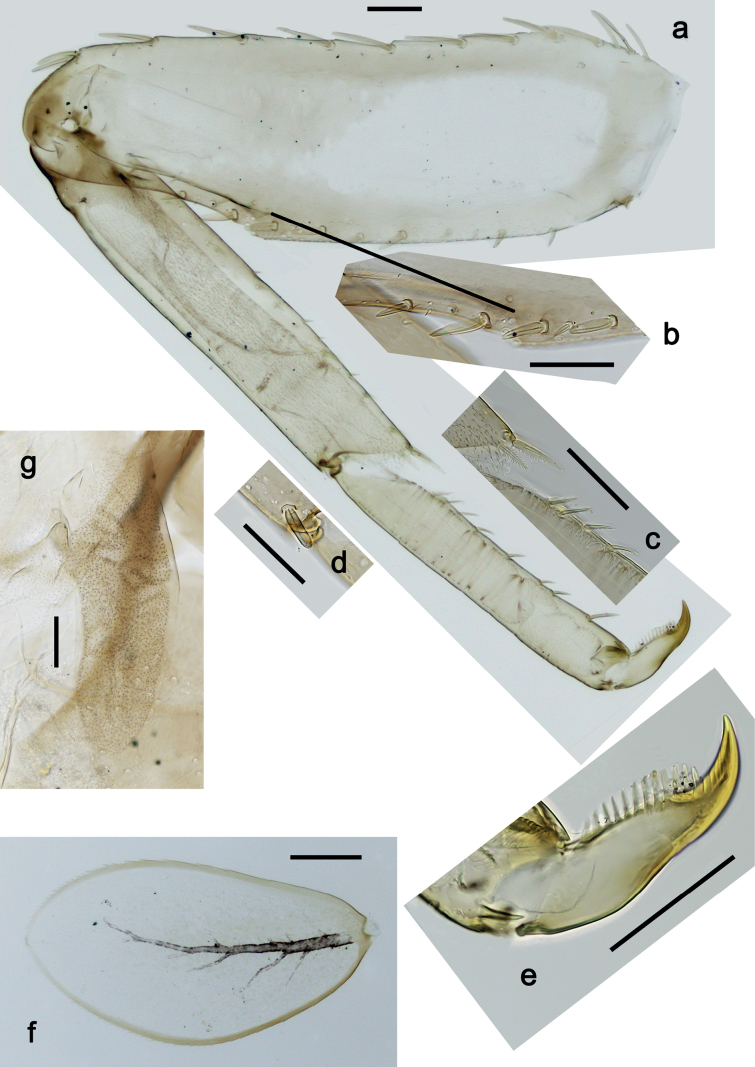
*Nigrobaetissuma* sp. nov., larva morphology **a** foreleg **b** setae on ventral margin of fore femur **c** ventral apex of fore tibia and base of fore tarsus **d** seta on dorsal apex of fore tibia **e** fore claw **f** tergalius IV **g** right hind protopteron. Scale bars: 50 µm.

**Figure 26.**
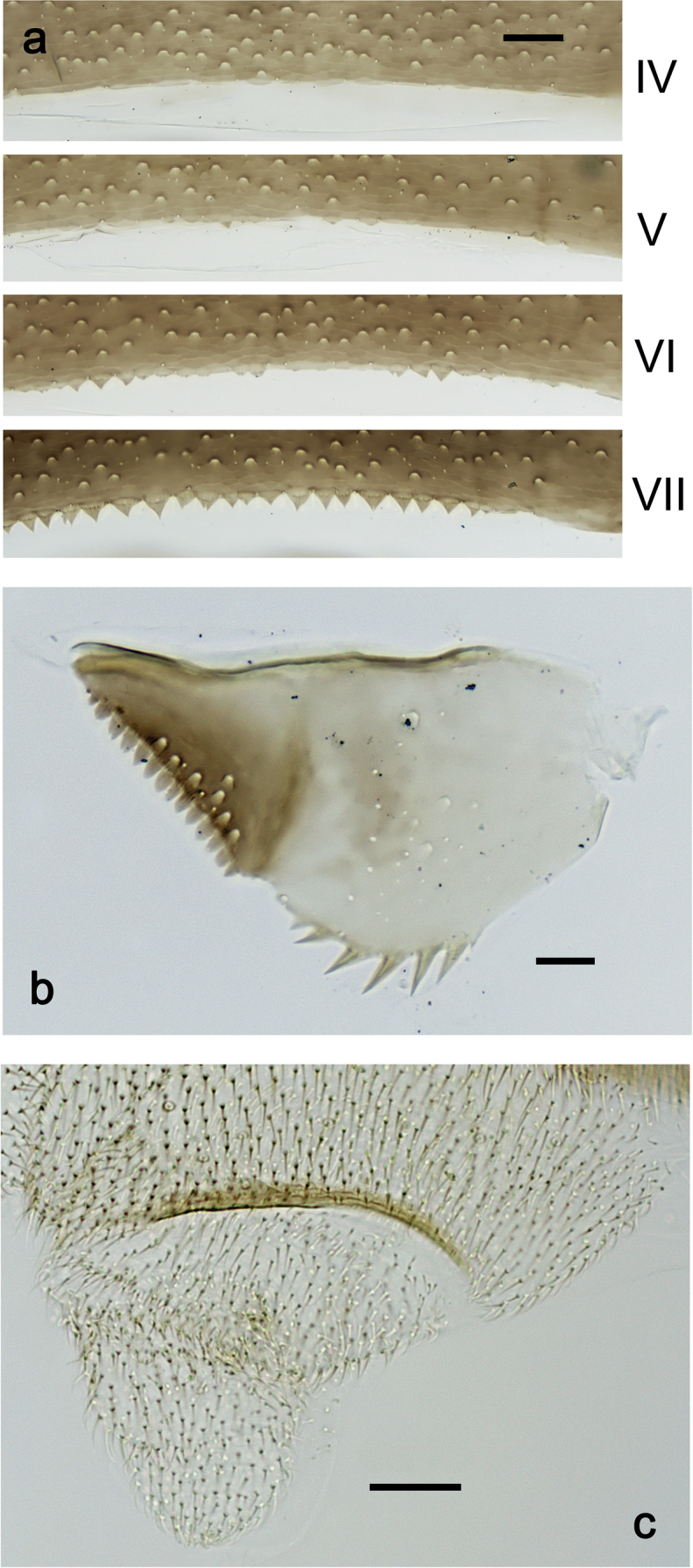
*Nigrobaetissuma* sp. nov., larva morphology **a** posterior margins of abdominal tergites IV–VII **b** paraproct **c** subimaginal gonostylus developing under cuticle of male last instar larva. Scale bars: 20 µm.

**Larva** (Figs 21–26). Body length 3.6–4.9 mm. Cerci ca. ¾ of body length, paracercus ca. ⅔ of cerci length. Antennae ca. 3× head length.

***Colouration*** (Fig. 21a, b). Head, thorax, and abdomen dorsally brown, abdominal terga VIII and IX bright beige. Head, thorax, and abdomen ventrally pale brown, abdominal sterna VIII–X ecru. Legs pale brown, femur basally, along margins, and in distal area darker. Caudalii pale brown.

***Labrum*** (Fig. 22a, b). Length 0.7× maximum width. Distal margin with medial emargination and a small process. Dorsally with medium, fine, simple setae scattered over surface; submedian seta and two long, simple, submarginal setae. Ventrally with marginal row of setae composed of lateral and anterolateral long, feathered setae and medial long, bifid, pectinate setae.

***Right mandible*** (Fig. 22c). Incisor and kinetodontium fused. Incisor with four denticles; kinetodontium with four denticles, inner margin of innermost denticle without row of thin setae. Prostheca stick-like, apicolaterally denticulate. Margin between prostheca and mola straight, with row of setae-like processes. Tuft of setae at apex of mola present.

***Left mandible*** (Fig. 22d). Incisor and kinetodontium fused. Incisor with four denticles; kinetodontium with three denticles. Prostheca robust, apically with small denticles and comb-shaped structure. Margin between prostheca and mola straight, with row of short, setae-like processes. Tuft of setae at apex of mola absent.

***Hypopharynx and superlinguae*** (Fig. 23a). Lingua approx. as long as superlinguae. Lingua longer than broad; medial tuft of stout setae poorly developed, broad; distal half laterally not expanded. Superlinguae distally straight; lateral margins rounded; fine, long, simple setae along distal margin.

***Maxilla*** (Fig. 23b–d). Galea-lacinia ventrally with two simple, apical setae under canines. Medially with one spine-like seta and ca. five long, simple setae. Maxillary palp ca. 1.3× as long as length of galea-lacinia; 2-segmented; segment II bent inwards, distal half sclerotised; palp segment II ca. 1.2× as long as segment I; setae on maxillary palp fine, simple, scattered over surface of segments I and II; apex of last segment slightly pointed.

***Labium*** (Fig. 24a–f). Glossa basally broad, narrowing toward apex; slightly shorter than paraglossa; inner margin with ca. eight spine-like setae; apex with two long and one medium, robust setae; outer margin with ca. six spine-like setae; ventral surface with fine, simple, scattered setae. Paraglossa curved inward; apex rounded; with three rows of long, robust, distally pectinate setae in apical area and ca. three medium, simple setae in medial and anteromedial area; dorsally with two long, spine-like, simple setae near inner margin. Labial palp with segment I 0.7× length of segments II and III combined. Segment I ventrally with short, fine, simple setae. Segment II without protuberance; ventral surface with short, fine, simple setae; dorsally with row of ca. three long, spine-like setae. Segment III subquadrangular; length ca. 0.9× maximum width; inner apical margin with some setae-like processes (Fig. 24f); ventrally with short, spine-like, simple setae and short, fine, simple setae.

***Hind protoptera*** (Fig. 25g) well developed.

***Foreleg*** (Fig. 25a–e). Ratio of foreleg segments 1.4:1.0:0.8:0.3. ***Femur*.** Length ca. 3× maximum width. Dorsal margin with ca. 11 curved, spine-like setae; length of setae 0.31× maximum width of femur. Apex rounded, with pair of spine-like setae. Many medium, stout, lanceolate setae along ventral margin; femoral patch absent. ***Tibia*.** Dorsal margin bare, on apex one stout, apically rounded seta. Ventral margin with row of short curved, spine-like setae, on apex two longer, spine-like, pectinate setae. Anterior surface with stout, lanceolate setae along ventral margin. Patellatibial suture present on basal half. ***Tarsus*.** Dorsal margin bare. Ventral margin with row of curved, spine-like setae. ***Claw*** with one row of 11 or 12 denticles; distally pointed; subapical setae absent.

***Middle and hind legs*.** As foreleg; *femur* ventrally with few stout, lanceolate setae; *tibia* dorsal margin with row of stout setae.

***Abdominal terga*** (Fig. 26a). Surface with irregular rows of U-shaped scale bases. Posterior margin of terga: I–IV(V) smooth, without spines; (V–)VI with rudimentary spines; VII–IX with triangular, pointed spines.

***Abdominal sterna*.** Posterior margin of sterna smooth, without spines.

***Tergalii*** (Fig. 25f). Present on segments I–VII. Margin with small denticles intercalating fine simple setae. Tracheae partly extending from main trunk toward inner and outer margins. Tergalius I as long as length of 2/3 of segment II; Tergalius IV as long as length of segments V and 1/3 VI combined; Tergalius VII as long as segments VIII and 1/3 IX combined.

***Paraproct*** (Fig. 26b). With six or seven stout, marginal spines. Surface scattered with U-shaped scale bases. Cercotractor with numerous small, marginal spines.

***Subimaginal gonostyli*** (Fig. 26c) developing under cuticle of male last instar larvae folded in *Nigrobaetis*-type (Kluge 2004: fig. 29G).

**Adult stages.** Unknown.

**Figure 27.**
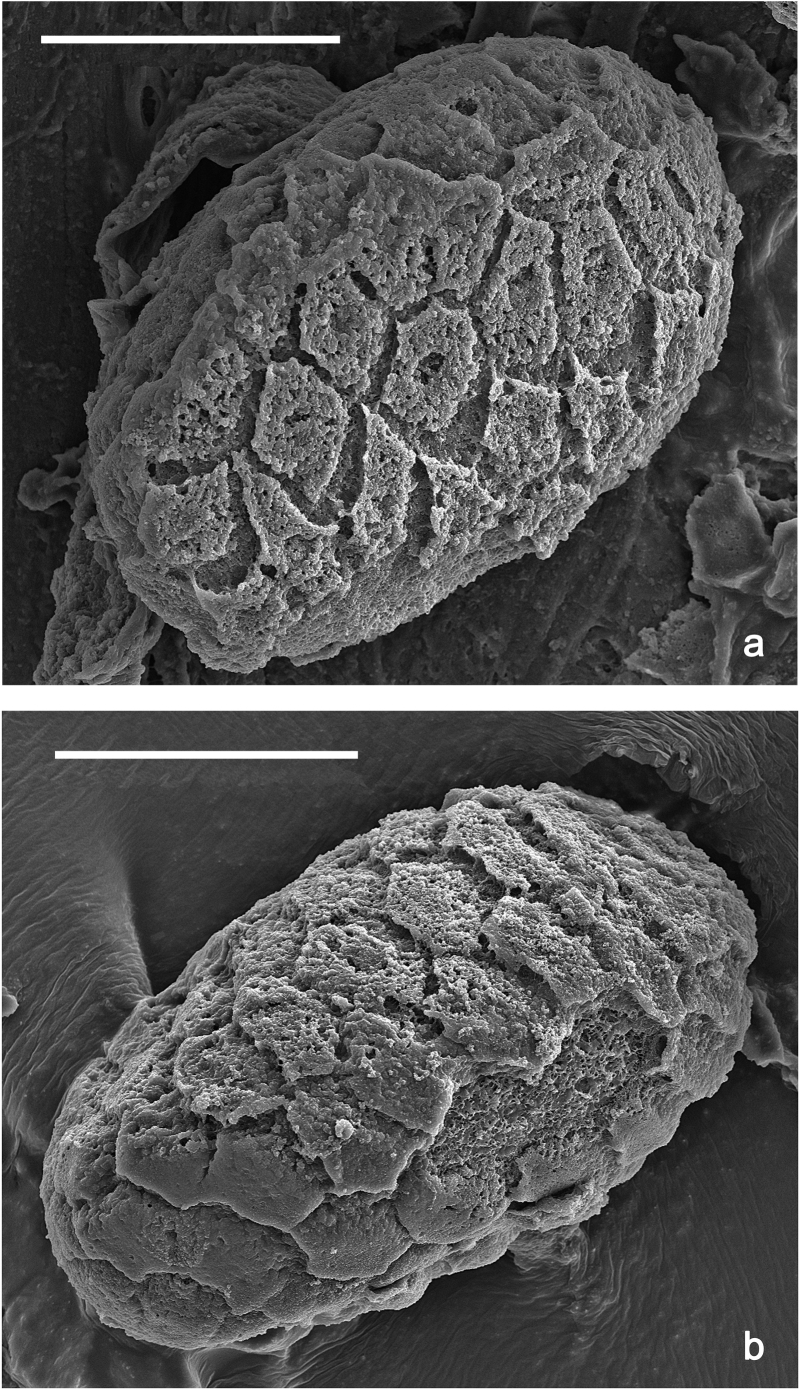
*Nigrobaetissuma* sp. nov., eggs. Scale bars: 40 µm.

**Eggs (Fig. 27).** Ovoid; surface with numerous polygonal structural elements (surface of the eggs in Fig. 27 could be partly degraded; a central, smaller, rounded, slightly elevated area surrounded by a round trench (as in other species) seems to be absent on the polygonal structural element, or invisible due to degradation).

###### Etymology.

Referring to the island of Sumatra, where the species was collected.

###### Distribution

**(Fig. 40c).** Indonesia, Sumatra.

###### Biological aspects.

The specimens were collected at altitudes from 10 m to 1300 m, mostly in deforested or agricultural areas. The streams were in average 2–5 m wide, 15–50 cm deep and flowing with 0.5–0.7 m/s. The substrate was always a mix of substantial amounts of boulder, stones, gravel, and sand.

###### Type-material.

***Holotype*.** Indonesia • larva; Sumatra, volcano Marapi, West; 00°28'29"S, 100°22'08"E; 553 m; 09.iv.2014; leg. M. Gueuning; on slide; GBIFCH00422020; MZL. ***Paratypes*.** Indonesia • 28 larvae; same data as holotype; 1 on slide; GBIFCH00592665; MZL; 27 in alcohol; GBIFCH00419696, GBIFCH00421961, GBIFCH00421976, GBIFCH00421985, GBIFCH00421992, GBIFCH00421997, GBIFCH00422012, GBIFCH00422014, GBIFCH00422016, GBIFCH00422017, GBIFCH00422034, GBIFCH00422435, GBIFCH00422704, GBIFCH00422929, GBIFCH00975675; MZL • 1 larva; Sumatra, volcano Marapi; 00°21'58"S, 100°33'18"E; 890 m; 02.iv.2014, leg. M. Gueuning; in alcohol; GBIFCH00422001; MZL • 1 larva; Sumatra, volcano Marapi, East; 00°21'33"S, 100°30'42"E; 1205 m; 03.iv.2014; leg. M. Gueuning; in alcohol; GBIFCH00422041; MZL • 2 larvae; Sumatra, volcano Sago, North, Riv. Simbukan; 00°17'08"S, 100°41'13"E; 880 m; 18.iii.2014; leg. M. Gueuning; in alcohol; GBIFCH00422027, GBIFCH00422037; MZL • 6 larvae; Sumatra, volcano Sago, South, Riv. Kobun; 00°22'33"S, 100°39'33"E; 1095 m; 19.iii.2014; leg. M. Gueuning; in alcohol; GBIFCH00421959, GBIFCH00421966, GBIFCH00422005, GBIFCH00422025, GBIFCH00422040, GBIFCH00423021; MZL • 2 larvae; Sumatra, volcano Sago, South, Riv. Tampo; 00°22'20"S, 100°41'45"E; 960 m; 20.iii.2014; leg. M. Gueuning; 1 on slide; GBIFCH00422032; 1 in alcohol; GBIFCH00421990; MZL • 1 larva; Sumatra, volcano Singgalang, riv. Caruak, 00°22'56"S, 100°22'42"E; 1300 m; 23.iii.2014; leg. M. Gueuning; in alcohol; GBIFCH00421956; MZL • 1 larva; Sumatra, volcano Singgalang, riv. Sianok; 00°19'57"S, 100°19'19"E; 1150 m; 24.iii.2014; leg. M. Gueuning; on slide; GBIFCH00422030; MZL • 10 larvae; Sumatra, volcano Singgalang, riv. Airjernih; 00°24'07"S, 100°16'44"E; 840 m; 25.iii.2014; leg. M. Gueuning; 1 on slide; GBIFCH00592666; 9 in alcohol; GBIFCH00421960, GBIFCH00421970, GBIFCH00422010, GBIFCH00422018, GBIFCH00422028, GBIFCH00422038, GBIFCH00422973; MZL • 6 larvae; Sumatra, volcano Talamau, South, Riv. Pularian; 00°00'60"N, 100°00'01"E; 540 m; 01.iv.2014; leg. M. Gueuning; 1 on slide; GBIFCH00422013; 5 in alcohol; GBIFCH00421981, GBIFCH00421965, GBIFCH00422004, GBIFCH00422021, GBIFCH00423066; MZL • 1 larva; Sumatra Barat, Sawahlunto, stream; 00°41'20"S, 100°46'43"E; 275.m; 10.xi.2011; leg. M. Balke; (UN5); in alcohol; GBIFCH00975705; MZL • 1 larva; Sumatra, upstream Tarusan; 10 m; 24.v.2010; leg. J.-M. Elouard; in alcohol; GBIFCH00975663; MZL • 1 larva; Sumatra, Batusangkar, Riv. Lupuak Tapuak; 440 m; 27.v.2010; leg. J.-M. Elouard; on slide; GBIFCH00975664; MZL.

##### ﻿ Nigrobaetis borneus sp. nov.

Taxon classificationAnimaliaEphemeropteraBaetidae

98DA648D-D534-5947-AE1A-9EE3F24DA6DB

https://zoobank.org/C934D238-B803-4B19-897C-C35BE989726E

**Figure 28.**
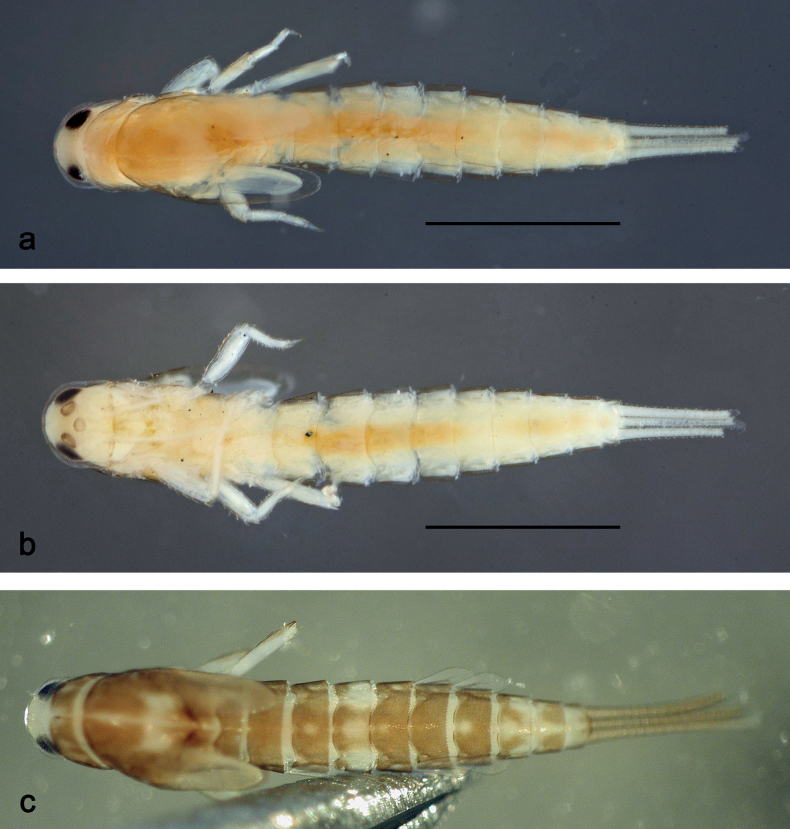
*Nigrobaetisborneus* sp. nov., larva habitus **a** dorsal view **b** ventral view (**a, b** after more than 20 years in 70% alcohol) **c** dorsal view (soon after collection). Scale bars: 1 mm.

**Figure 29.**
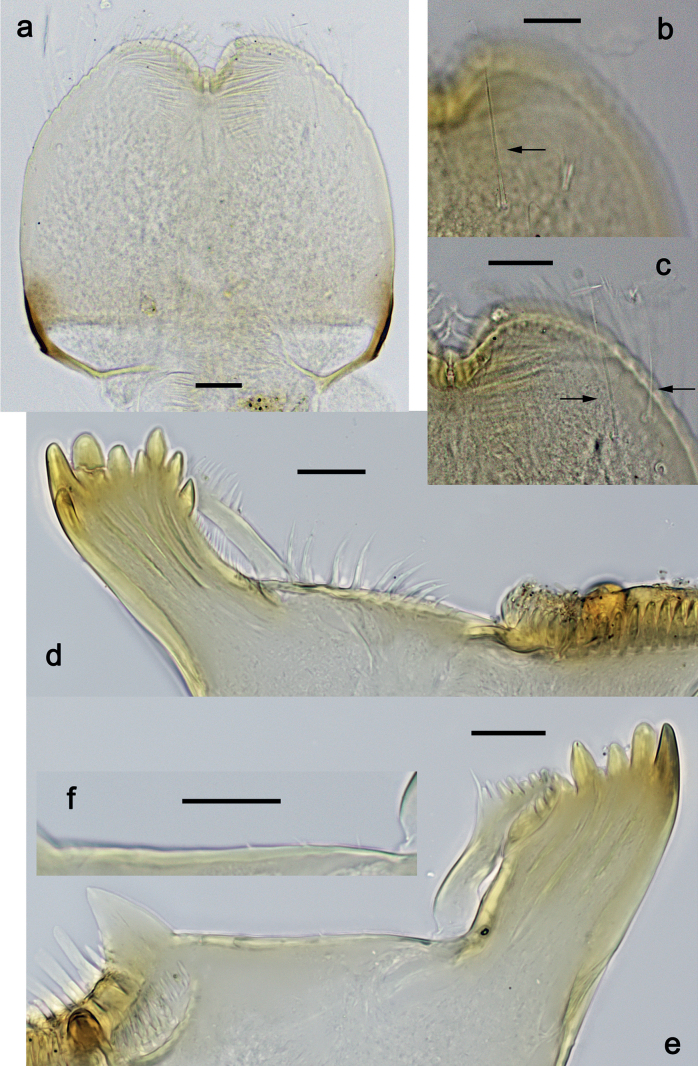
*Nigrobaetisborneus* sp. nov., larva morphology **a** labrum, ventral focus **b** labrum, dorsal focus (arrow: submedian seta) **c** labrum, dorsal focus (arrows: submarginal setae) **d** right mandible **e** left mandible **f** left mandible, margin between prostheca and mola. Scale bars: 20 µm.

**Figure 30.**
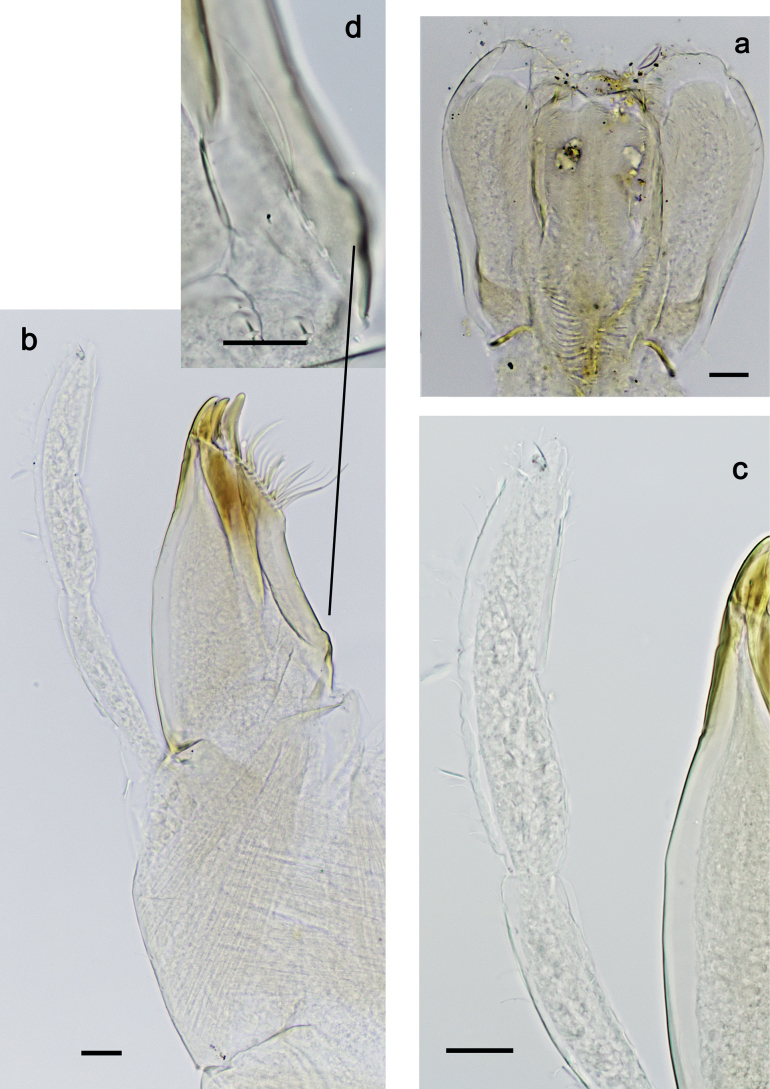
*Nigrobaetisborneus* sp. nov., larva morphology **a** hypopharynx and superlinguae**b** maxilla **c** maxillary palp **d** maxilla, ventrolateral section. Scale bars: 20 µm.

**Figure 31.**
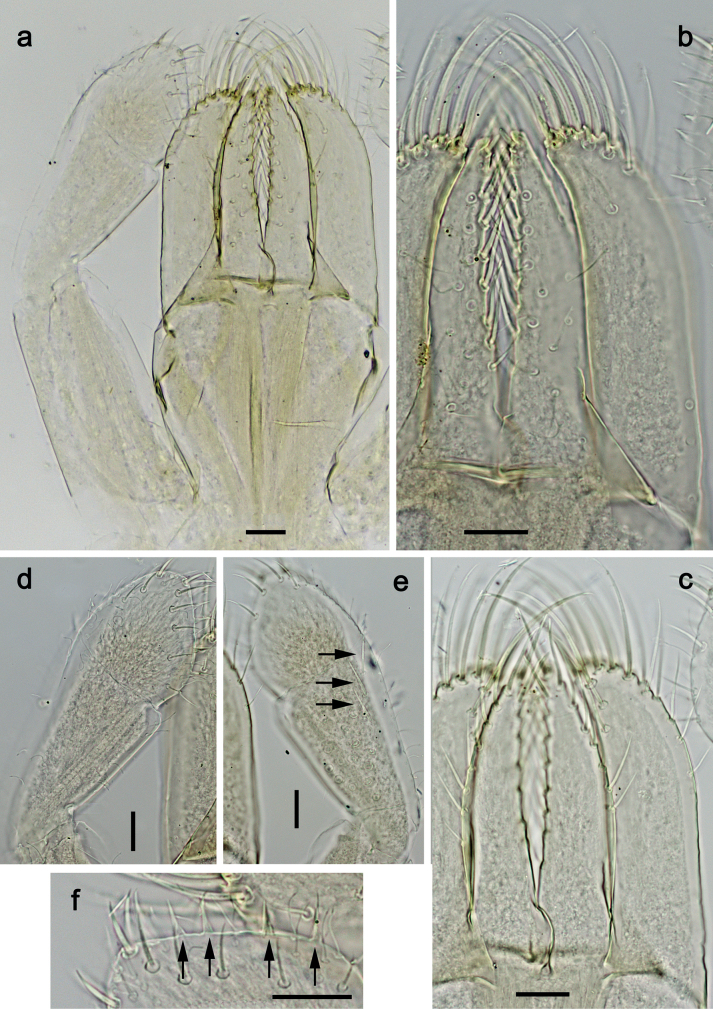
*Nigrobaetisborneus* sp. nov., larva morphology **a** labium **b** glossae and paraglossae, ventral focus **c** glossae and paraglossae, dorsal focus **d** labial palp, ventral focus **e** labial palp, dorsal focus **f** labial palp, inner apical margin of segment III (arrows: setae-like processes). Scale bars: 20 µm.

**Figure 32.**
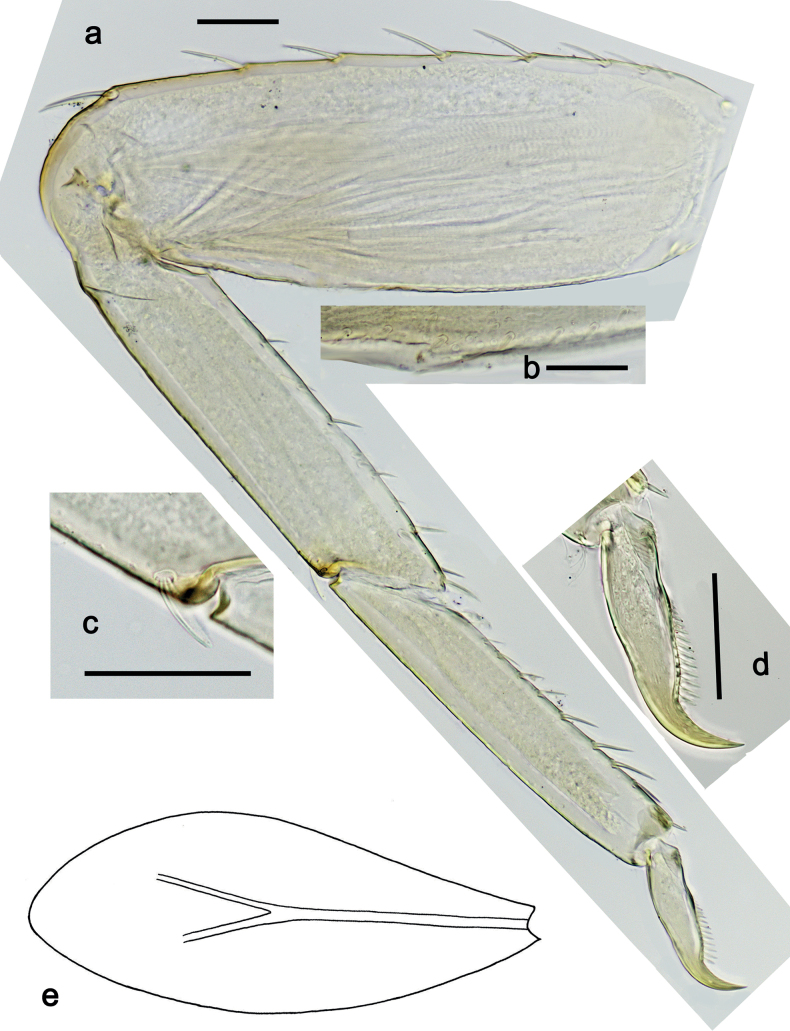
*Nigrobaetisborneus* sp. nov., larva morphology **a** foreleg **b** setae on ventral margin of fore femur **c** seta on dorsal apex of fore tibia **d** fore claw **e** tergalius V. Scale bars: 50 µm.

**Figure 33.**
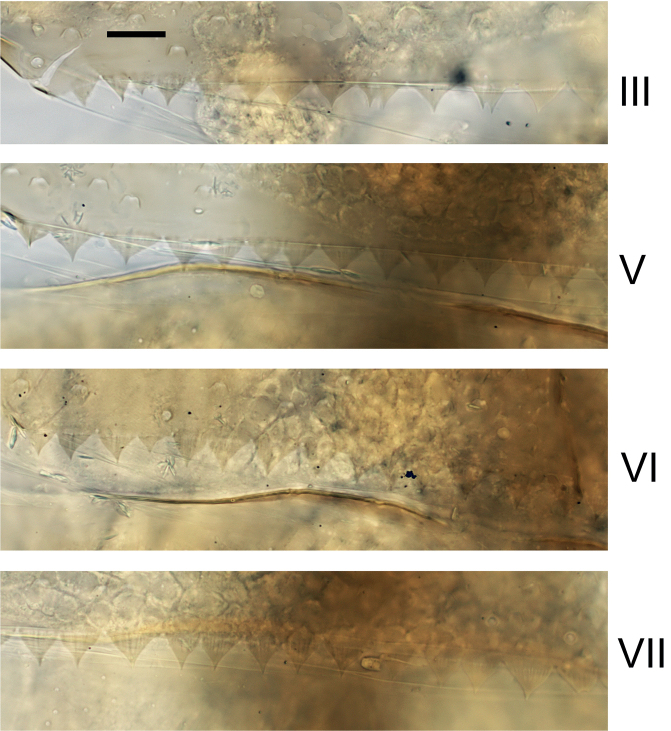
*Nigrobaetisborneus* sp. nov., larva morphology a posterior margins of abdominal tergites III, V–VII. Scale bars: 20 µm.

Figs 28 , 29 , 30 , 31 , 32 , 33 , 40c

###### Differential diagnosis.

**Larva.** Following combination of characters: A) dorsal surface of labrum with submedian seta and two long, simple setae in submarginal position; length 0.8× width (Fig. 29a–c); B) right mandible: incisor with five denticles, kinetodontium with three denticles (Fig. 29d); C) left mandible: incisor with four denticles, kinetodontium with four denticles; margin between prostheca and mola straight, with row of few short, setae-like processes (Fig. 29e); D) fore femur length ca. 2.4× maximum width, dorsal margin with 7–9 curved, spine-like setae (Fig. 32a); E) tibia dorsally bare, distally with one stout, apically rounded seta (Fig. 32a, c); F) claw with 12–14 denticles (Fig. 32d); G) hind protoptera absent; H) tergalii on abdominal segments I–VII; I) posterior margins of abdominal terga: I smooth, without spines; II–IX with triangular, pointed spines (Fig. 33).

###### Description.

**Larva** (Figs 28–33). Body length 3.0–3.3 mm. Caudalii broken. Antennae ca. 2.5× head length.

***Colouration*** (Fig. 28a–c). Head dorsally pale brown, thorax, and abdomen dorsally brown, with bright pattern as Fig. 28c. Head, thorax, and abdomen ventrally pale brown. Legs pale brown, femur apically brown and with a brown, distomedial spot. Caudalii brown.

***Labrum*** (Fig. 29a–c). Length 0.8× maximum width. Distal margin with medial emargination and a small process. Dorsally with medium, fine, simple setae scattered over surface; submedian seta and two long, simple, submarginal setae. Ventrally with marginal row of setae composed of anterolateral long, feathered setae and medial long, bifid, pectinate setae.

***Right mandible*** (Fig. 29d). Incisor and kinetodontium fused. Incisor with four denticles; kinetodontium with five denticles, inner margin of innermost denticle without row of thin setae. Prostheca stick-like, apicolaterally denticulate. Margin between prostheca and mola straight, with row of setae-like processes. Tuft of setae at apex of mola present.

***Left mandible*** (Fig. 29e). Incisor and kinetodontium fused. Incisor with four denticles; kinetodontium with four denticles. Prostheca robust, apically with small denticles and comb-shaped structure. Margin between prostheca and mola straight, with row of few short, setae-like processes. Tuft of setae at apex of mola absent.

***Hypopharynx and superlinguae*** (Fig. 30a). Lingua shorter than superlinguae. Lingua longer than broad; medial tuft of stout setae poorly developed; distal half laterally not expanded. Superlinguae distally rounded; lateral margins rounded; fine, long, simple setae along distal margin.

***Maxilla*** (Fig. 30b–d). Galea-lacinia ventrally with two simple, apical setae under canines. Medially with one spine-like seta and ca. four long, simple setae. Maxillary palp ca. 1.2× as long as length of galea-lacinia; 2-segmented; palp segment II ca. 1.3× as long as segment I; setae on maxillary palp fine, simple, scattered over surface of segments I and II; apex of last segment slightly pointed.

***Labium*** (Fig. 31a–f). Glossa basally broad, narrowing toward apex; approx. as long as paraglossa; inner margin with ca. eight spine-like setae; apex with two long and one medium, robust setae; outer margin with ca. six spine-like setae; ventral surface with fine, simple, scattered setae. Paraglossa curved inward; apex rounded; with three rows of long, robust, distally pectinate setae in apical area and ca. two medium, simple setae in anteromedial area; dorsally with three long, spine-like, simple setae near inner margin. Labial palp with segment I 0.9× length of segments II and III combined. Segment I ventrally with short, fine, simple setae. Segment II without protuberance; ventral surface with short, fine, simple setae; dorsally with row of ca. three long, spine-like setae. Segment III slightly pentagonal; length subequal to maximum width; inner apical margin with some setae-like processes (Fig. 31f); ventrally with short, spine-like, simple setae and short, fine, simple setae.

***Hind protoptera***: Absent.

***Foreleg*** (Fig. 32a–d). Ratio of foreleg segments 1.2:1.0:0.9:0.3. ***Femur*.** Length ca. 2.4× maximum width. Dorsal margin with 7–9 curved, spine-like setae; length of setae 0.27× maximum width of femur. Apex rounded, with pair of spine-like setae. Medium, stout, lanceolate setae along ventral margin; femoral patch absent. ***Tibia*.** Dorsal margin bare, on apex one stout, apically rounded seta. Ventral margin with row of short to medium curved, spine-like setae, on apex two longer, spine-like, pectinate setae. Anterior surface with few stout, lanceolate setae along ventral margin. Patellatibial suture present on basal half. ***Tarsus*.** Dorsal margin bare. Ventral margin with row of curved, spine-like setae. ***Claw*** with one row of 12–14 denticles; distally pointed; subapical setae absent.

***Middle and hind legs*.** As foreleg.

***Abdominal terga*** (Fig. 33). Surface with irregular rows of U-shaped scale bases. Posterior margin of terga: I smooth, without spines; II–IX with triangular, pointed spines.

***Abdominal sterna*.** Posterior margin of sterna smooth, without spines.

***Tergalii*** (Fig. 32e). Present on segments I–VII. Margin with small denticles intercalating fine simple setae. Tracheae restricted to main trunk and few branches, not reaching margins. Tergalius I as long as length of segment II; Tergalius IV as long as length of segments V and 1/2 VI combined; Tergalius VII as long as segments VIII and 1/2 IX combined.

***Paraproct*.** With six or seven stout, marginal spines. Surface scattered with U-shaped scale bases, fine, simple setae, and micropores. Cercotractor with numerous small, marginal spines.

**Adult stages.** Unknown.

###### Etymology.

Referring to the island of Borneo, where the species was collected.

###### Distribution

**(Fig. 40c).** Indonesia, Borneo (East Kalimantan).

###### Biological aspects.

The specimens were collected at an altitude of 160 m in a large (width ca. 36 m), shallow (depth ca. 0.1 m), fast flowing (ca. 0.9 m/s) stream. The stream was characterised by equally run/riffles and pools. Water temperature was 25 °C, substrate was dominated by boulder and cobble.

###### Type-material.

***Holotype*.** Indonesia • larva; East Kalimantan, Bas. Malinau, riv. Seturan, loc. Seturan (2001-block 57), trib. Bengahau; 02°59'22"N, 116°30'46"E; 08.viii.2000; leg. P. Derleth; on slide; GBIFCH00592663; MZL. ***Paratypes*.** Indonesia • 3 larvae; same data as holotype; 1 on slide; GBIFCH00975692; 2 in alcohol; GBIFCH00975674; MZL.

##### ﻿ Nigrobaetis kaliman sp. nov.

Taxon classificationAnimaliaEphemeropteraBaetidae

2A501767-DAB2-52D3-A9C7-D9E79A219EAB

https://zoobank.org/6B919B4B-AE54-40FB-9951-BF0C40797BD9

**Figure 34.**
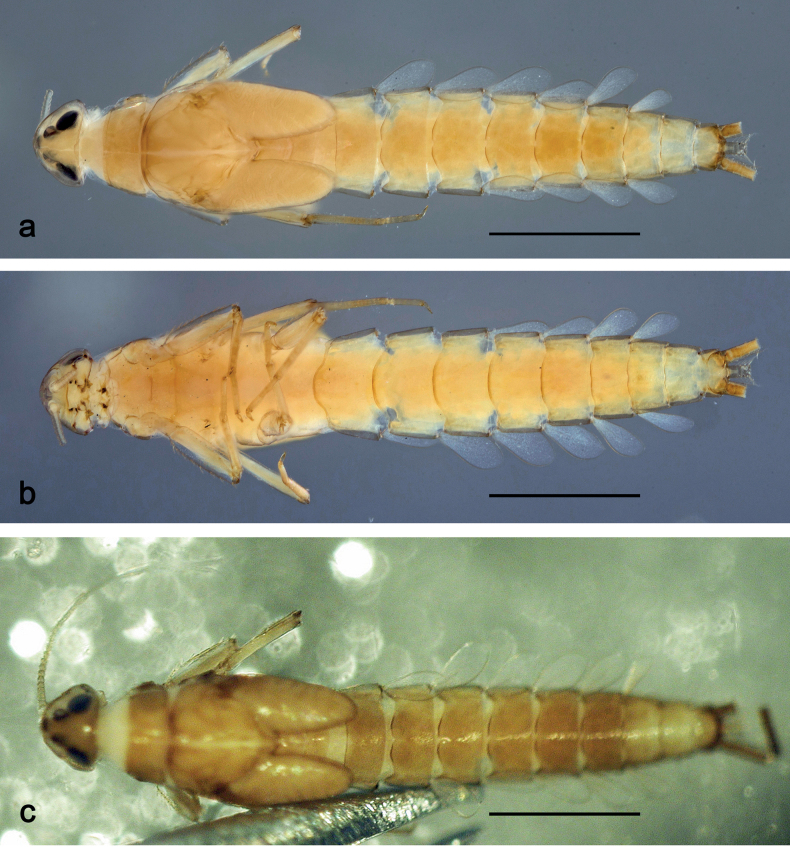
*Nigrobaetiskaliman* sp. nov., larva habitus **a** dorsal view **b** ventral view (**a, b** after more than 20 years in 70% alcohol) **c** dorsal view (soon after collection). Scale bars: 1 mm.

**Figure 35.**
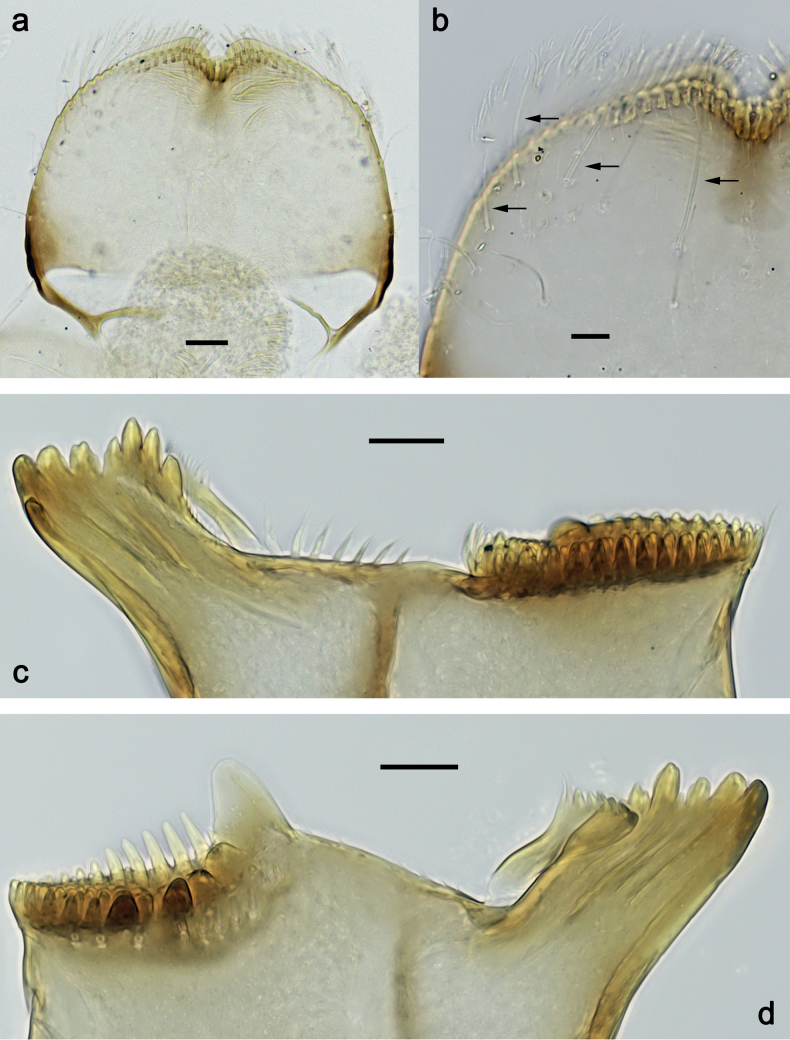
*Nigrobaetiskaliman* sp. nov., larva morphology **a** labrum, ventral focus **b** labrum, dorsal focus (arrows: submedian and submarginal setae) **c** right mandible **d** left mandible. Scale bars: 20 µm.

**Figure 36.**
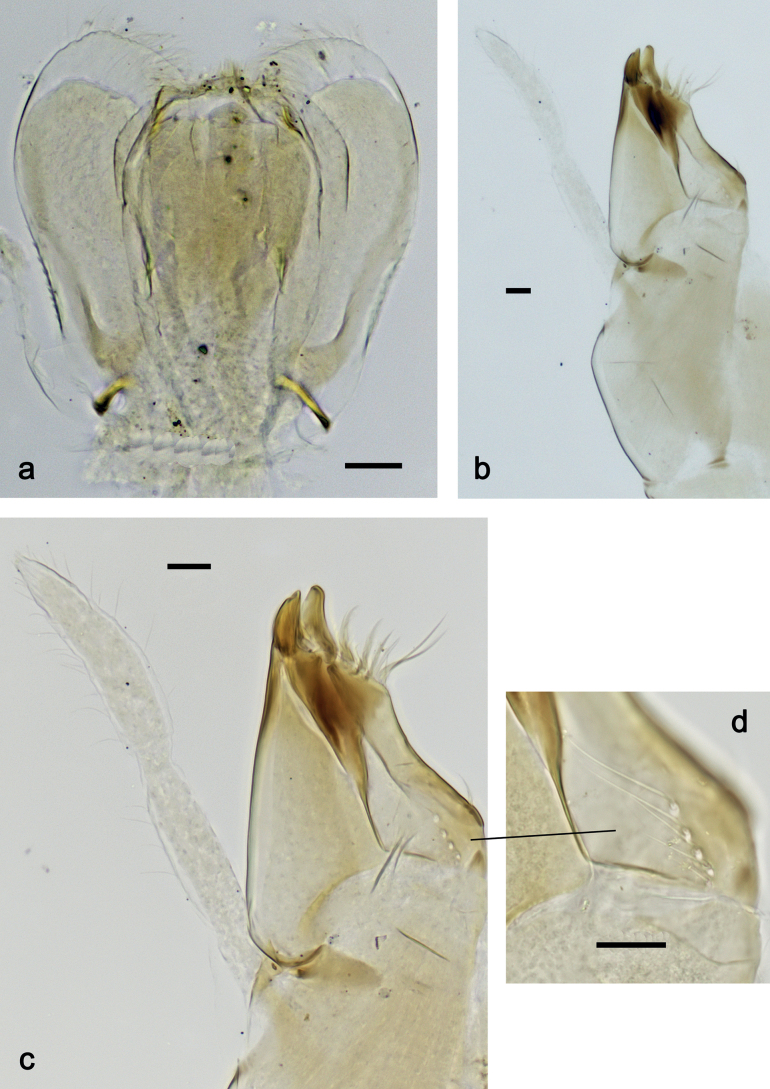
*Nigrobaetiskaliman* sp. nov., larva morphology **a** hypopharynx and superlinguae**b, c** maxilla **d** maxilla, ventrolateral section. Scale bars: 20 µm.

**Figure 37.**
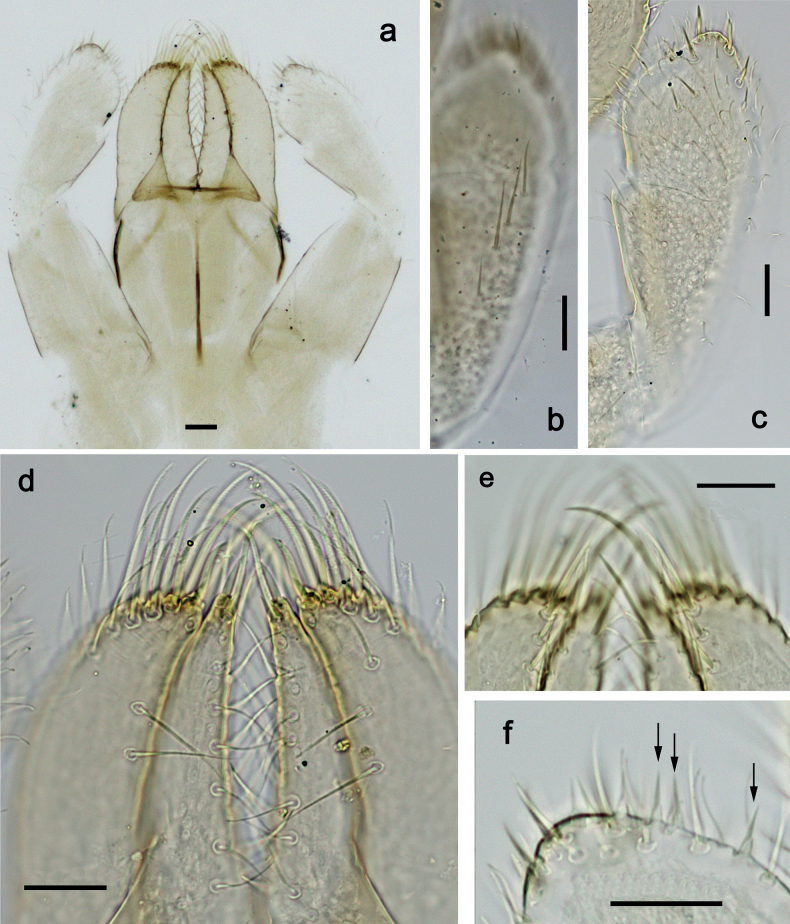
*Nigrobaetiskaliman* sp. nov., larva morphology **a** labium **b** labial palp, dorsal focus **c** labial palp, ventral focus **d** glossae and paraglossae, ventral focus **e** apex of glossae and paraglossae, dorsal focus **f** labial palp, inner apical margin of segment III (arrows: setae-like processes). Scale bars: 20 µm.

**Figure 38.**
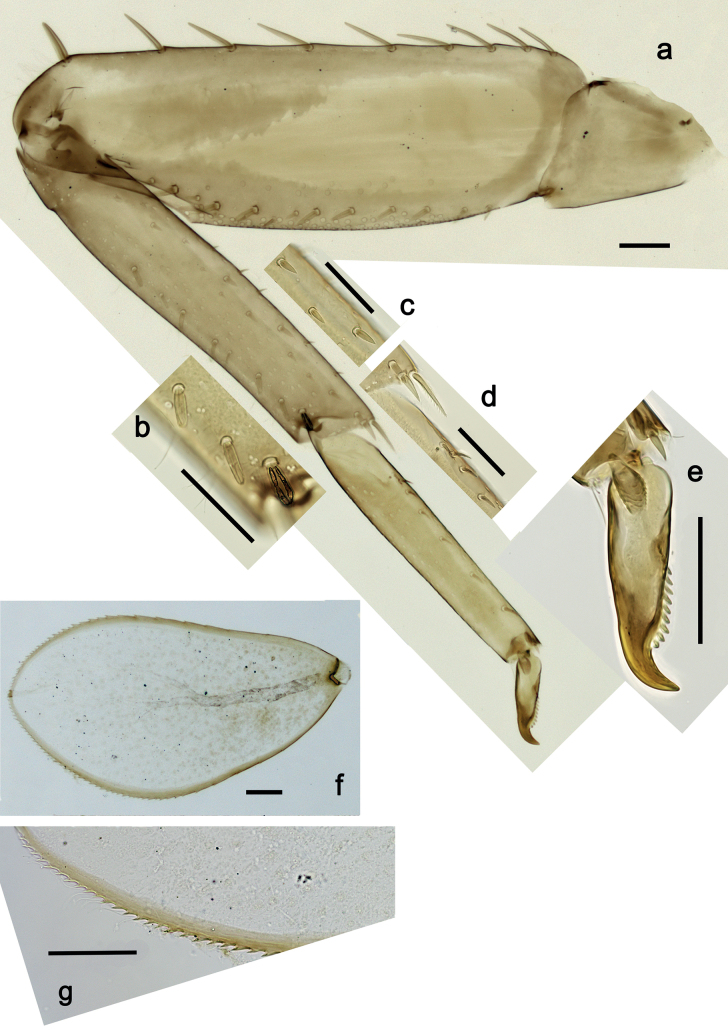
*Nigrobaetiskaliman* sp. nov., larva morphology **a** foreleg **b** setae on dorsal apex of fore tibia **c** setae of anterior surface of fore tibia **d** ventral apex of fore tibia and base of fore tarsus **e** fore claw **f** tergalius IV **g** section of margin of tergalius IV. Scale bars: 50 µm.

**Figure 39.**
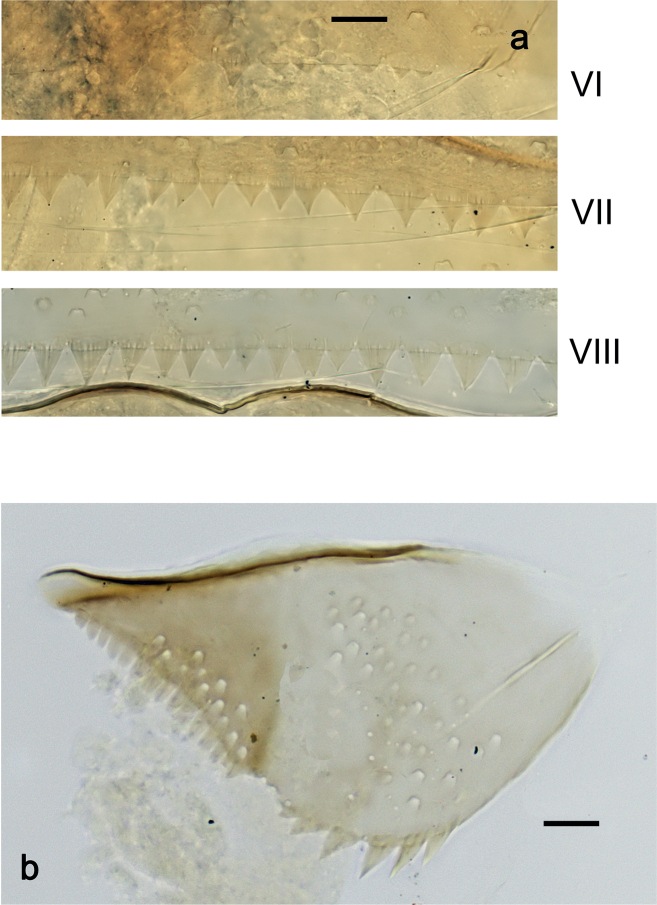
*Nigrobaetiskaliman* sp. nov., larva morphology **a** posterior margins of abdominal tergites VI–VII **b** paraproct. Scale bars: 20 µm.

**Figure 40.**
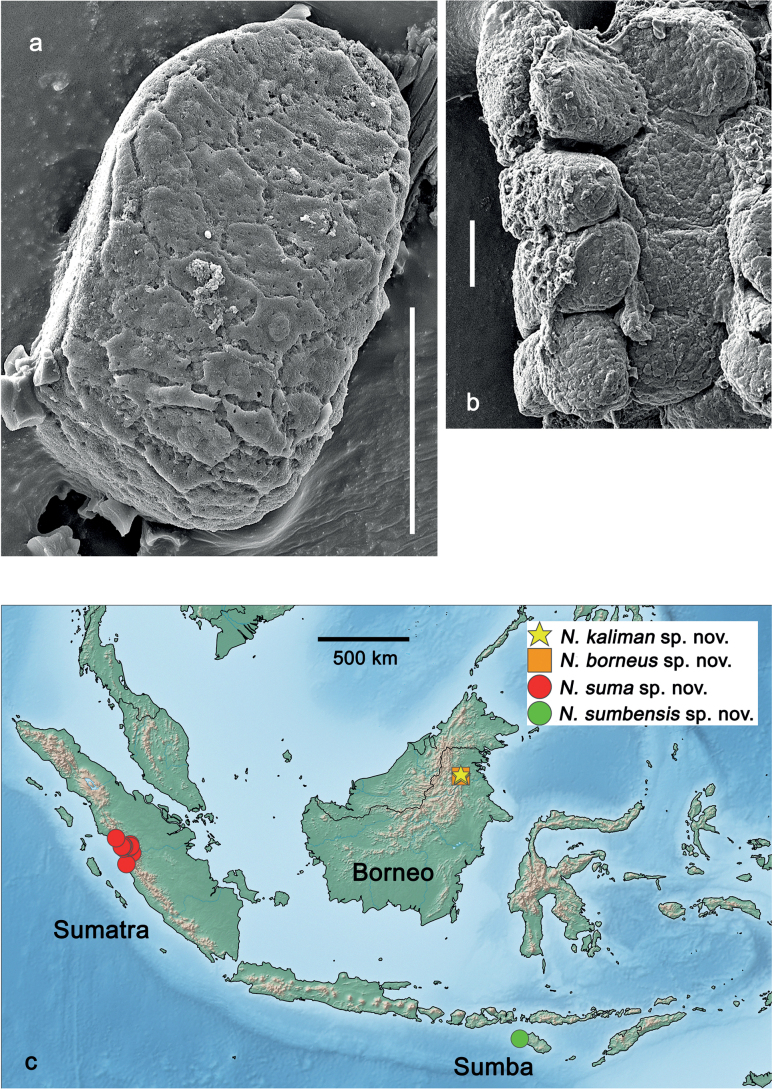
**a***Nigrobaetiskaliman* sp. nov., egg **b***Nigrobaetiskaliman* sp. nov., pack of developing eggs (extracted from same larva than a) **c** distribution of *Nigrobaetis* species in Indonesia. Scale bars: 40 µm (**a, b**).

Figs 34 , 35 , 36 , 37 , 38 , 39 , 40

###### Differential diagnosis.

**Larva.** Following combination of characters: A) dorsal surface of labrum with submedian seta and a short, submarginal arc of three long, simple setae (Fig. 35b); B) right mandible: incisor with four denticles, kinetodontium with four denticles (Fig. 35c); C) left mandible: incisor with four or five denticles, kinetodontium with three denticles; margin between prostheca and mola straight, with row of few short, setae-like processes (Fig. 35d); D) fore femur length ca. 3× maximum width, dorsal margin with 7–9 curved, spine-like setae (Fig. 38a); E) tibia dorsally with row of spine-like, apically rounded setae; distally with one stout, apically rounded seta (Fig. 38a, b); F) claw with ten or eleven denticles (Fig. 38e); G) hind protoptera absent; H) tergalii on abdominal segments I–VII; I) posterior margins of abdominal terga: I–V (IV) smooth, without spines; (V) VI–IX with triangular, pointed spines (Fig. 39a).

###### Description.

**Larva** (Figs 34–39). Body length 3.5–4.6 mm. Caudalii broken. Antennae ca. 3× head length.

***Colouration*** (Fig. 34a–c). Head, thorax, and abdomen dorsally brown, abdominal terga VI and VII darker, abdominal terga VIII and IX pale brown. Head, thorax, and abdomen ventrally pale brown. Legs brown, femur mediobasally with bright area. Caudalii brown.

***Labrum*** (Fig. 35a, b). Length 0.7× maximum width. Distal margin with medial emargination and a small process. Dorsally with medium, fine, simple setae scattered over surface; submedian seta and several long, simple, submarginal setae. Ventrally with marginal row of setae composed of anterolateral long, feathered setae and medial long, bifid, pectinate setae.

***Right mandible*** (Fig. 35c). Incisor and kinetodontium fused. Incisor with four denticles; kinetodontium with four denticles, inner margin of innermost denticle without row of thin setae. Prostheca stick-like, apicolaterally denticulate. Margin between prostheca and mola straight, with row of setae-like processes. Tuft of setae at apex of mola present.

***Left mandible*** (Fig. 35d). Incisor and kinetodontium fused. Incisor with four denticles; kinetodontium with three denticles. Prostheca robust, apically with small denticles and comb-shaped structure. Margin between prostheca and mola straight, with row of short, setae-like processes. Tuft of setae at apex of mola absent.

***Hypopharynx and superlinguae*** (Fig. 36a). Lingua shorter than superlinguae. Lingua longer than broad; medial tuft of stout setae poorly developed; distal half laterally slightly expanded. Superlinguae distally rounded; lateral margins rounded; fine, long, simple setae along distal margin.

***Maxilla*** (Fig. 36b–d). Galea-lacinia ventrally with two simple, apical setae under canines. Medially with one spine-like, pectinate seta and ca. five long, simple setae. Maxillary palp ca. 1.3× as long as length of galea-lacinia; 2-segmented; palp segment II approx. as long as segment I; setae on maxillary palp fine, simple, scattered over surface of segments I and II; apex of last segment slightly pointed.

***Labium*** (Fig. 37a–f). Glossa basally broad, narrowing toward apex; approx. as long as paraglossa; inner margin with ca. seven spine-like setae; apex with two long and one medium, robust setae; outer margin with ca. six spine-like setae; ventral surface with row of fine, simple, setae along inner margin. Paraglossa curved inward; apex rounded; with three rows of long, robust, distally pectinate setae in apical area and ca. three medium, simple setae in distal area; dorsally with three long, spine-like, simple setae near inner margin. Labial palp with segment I slightly longer than segments II and III combined. Segment I ventrally with short, fine, simple setae. Segment II without protuberance; ventral surface with short, fine, simple setae; dorsally with row of ca. four long, spine-like setae. Segment III slightly pentagonal; length ca. 1.2× maximum width; inner apical margin with some setae-like processes (Fig. 37f); ventrally with short, spine-like, simple setae and short, fine, simple setae.

***Hind protoptera***: Absent.

***Foreleg*** (Fig. 38a–e). Ratio of foreleg segments 1.3:1.0:0.7:0.2. ***Femur*.** Length ca. 3× maximum width. Dorsal margin with 7–9 curved, spine-like setae; length of setae 0.31× maximum width of femur. Apex rounded, with pair of spine-like setae. Medium, stout, lanceolate setae along ventral margin; femoral patch absent. ***Tibia*.** Dorsal margin with row of spine-like, apically rounded setae; on apex one stout, apically rounded seta. Ventral margin with row of short, curved, spine-like setae; on apex two longer, spine-like, pectinate setae. Anterior surface with stout, lanceolate, slightly pectinate setae along ventral margin. Patellatibial suture present on basal half. ***Tarsus*.** Dorsal margin bare. Ventral margin with row of curved, spine-like, pectinate setae. ***Claw*** with one row of ten or eleven denticles; distally pointed; subapical setae absent.

***Middle and hind legs*.** As foreleg.

***Abdominal terga*** (Fig. 39a). Surface with irregular rows of U-shaped scale bases. Posterior margin of terga: I–V (IV) smooth, without spines; (V) VI–IX with triangular, pointed spines.

***Abdominal sterna*.** Posterior margin of sterna smooth, without spines.

***Tergalii*** (Fig. 38f). Present on segments I–VII. Margin with small denticles intercalating fine simple setae. Tracheae restricted to main trunk and few branches, not reaching margins. Tergalius I as long as length of segment II; Tergalius IV as long as length of segments V and 1/3 VI combined; Tergalius VII as long as segments VIII and IX combined.

***Paraproct*.** With six or seven stout, marginal spines. Surface scattered with U-shaped scale bases and micropores. Cercotractor with numerous small, marginal spines.

**Adult stages.** Unknown.

**Eggs (Fig. 40a, b).** Egg of Fig. 40a still in early stage of development, probably not showing the final shape (see Fig. 40b, pack of developing eggs extracted from same larva). Surface with numerous papillae-like structural elements (polygonal to rounded structure, centrally with a smaller, rounded, slightly elevated area surrounded by a round trench).

###### Etymology.

Referring to the province Kalimantan (Indonesia, Borneo), where the species was collected.

###### Distribution

**(Fig. 40c).** Indonesia, Borneo (East Kalimantan).

###### Biological aspects.

The specimens were collected on altitudes of ca. 130 m. The streams were 2–30 m wide, ca. 0.1 m deep, and flowing with ca. 0.6 m/s. The location was dominated by riffles and run, substrate was mainly gravel and cobble, water temperature ca. 26 °C.

###### Type-material.

***Holotype*.** Indonesia • larva; East Kalimantan, Bas. Malinau, riv. Seturan, loc. Seturan, main river; 116°30'48"E, 03°00'05"N, 28.iii.2001; leg. P. Derleth and B. Feldmeyer; on slide; GBIFCH00592661; MZL. ***Paratypes*.** Indonesia • 3 larvae; same data as holotype; 2 on slides; GBIFCH00592655, GBIFCH00592662; MZL • 2 larvae; East Kalimantan, Bas. Malinau, Riv. Seturan, Loc. Seturan (1999-bloc 39–40), Trib. Temalat (Sungai Guang); 03°00'10"N, 116°32'24"E; 27.iii.2001; leg. P. Derleth; in alcohol; GBIFCH00975671; MZL • 2 larvae; East Kalimantan, Bas. Malinau, Riv. Seturan, Loc. Seturan (2001-bloc 57), Trib. Tamalang (Sungai Guang); 18.vii.2001; leg. P. Derleth and F. Béboux; 1 on slide; GBIFCH00592614; 1 in alcohol; GBIFCH00975683; MZL • 5 larvae; East Kalimantan, Bas. Malinau, Riv. Seturan, Loc. Seturan (2001-bloc 57), Trib. Tamalang (Sungai Guang); 10.iv.2001; leg. P. Derleth; in alcohol; GBIFCH00975684, GBIFCH00975685; MZL • 2 larvae; East Kalimantan, Bas. Malinau, riv. Rian, loc. Langap South (1997-bloc 6), trib. Belakau; 116°30'26"E, 3°04'04"N; 18.iv.2001; leg. P. Derleth and M. Sartori; in alcohol; GBIFCH00975672; MZL • 1 larva; East Kalimantan, Bas. Malinau, riv. Seturan, loc. Seturan (2000-bloc 43), trib. Temalat (Sungai Guang); 116°33'29"E, 02°59'29"N; 16.viii.2000; leg. P. Derleth and R. Schlaepfer; in alcohol; GBIFCH00975673; MZL • 3 larvae; East Kalimantan, Bas. Malinau, Riv. Rian, Loc. Langap Sud (1995), Trib. Ngayo; 03°04'56"N, 116°30'58"E; 17.iv.2001; leg. P. Derleth and M. Sartori; 2 on slides; GBIFCH00592656, GBIFCH00975691; 1 in alcohol; GBIFCH00975693; MZL.

### ﻿Key to *Nigrobaetis* species (larvae) of Indonesia, the Philippines, and neighbouring continental Southeast Asia

**Table:** 

1	Hind protoptera well developed (Fig. 25g)	**2**
–	Hind protoptera absent or minute	**4**
2	Paraproct with > 30 marginal spines (Müller-Liebenau 1984: fig. 5i)	***N.gombaki***
–	Paraproct with < 10 marginal spines (Fig. 26b)	**3**
3	Dorsal margin of fore femur with ca. 7 spine-like setae and some shorter additional setae along margin (Müller-Liebenau 1984: fig. 4j); paraproct with ca. 4 marginal spines (Müller-Liebenau 1984: fig. 4i); left mandible with bare margin between prostheca and mola (Müller-Liebenau 1984: fig. 4e)	***N.mirabilis***
–	Dorsal margin of fore femur with ca. 11 spine-like setae and without additional setae along margin (Fig. 25a); paraproct with 6 or 7 marginal spines (Fig. 26b); left mandible with row of short, setae-like processes on margin between prostheca and mola (Fig. 22c)	***N.suma* sp. nov.**
4	Fore femur very slender (length to maximum width ca. 4.8×), dorsally slightly concave (Fig. 19a); claw with 14–18 denticles (Fig. 19c); left mandible with tuft of medium setae-like processes between prostheca and mola (Fig. 16d)	***N.sumbensis* sp. nov.**
–	Fore femur rather broad (length to maximum width ca. 2–3×), dorsally straight (Fig. 5a); claw with not more than 14 denticles (usually 8–12); left mandible with partial or complete row of short, setae-like processes between prostheca and mola, or bare (Figs 2d, 10d)	**5**
5	Fore tibia dorsal margin with row of medium, spine-like, apically rounded setae (Fig. 38a, b)	***N.kaliman* sp. nov.**
–	Fore tibia dorsal margin almost bare (sometimes with 1–3 short, spine-like setae and usually with stout, apically rounded seta at apex) (Fig. 32a, c)	**6**
6	With minute hind protoptera (Fig. 5e); labial palp segment III apically with rounded protrusion, length subequal to width (Fig. 4a, c)	***N.plures* sp. nov.**
–	Hind protoptera absent; labial palp segment III without apical protrusion, longer than maximal width (Fig. 12a, c)	**7**
7	Posterior margin of tergum IV with triangular spines, longer than wide (Müller-Liebenau 1984: fig. 35)	***N.minutus***
–	Posterior margin of tergum IV with triangular spines, wider than long (Fig. 14a)	**8**
8	Labrum relatively long (length 0.8× width) (Fig. 29a); margin between prostheca and mola of left mandible with row of minute setae-like processes (Fig. 29e, f)	***N.borneus* sp. nov.**
–	Labrum of usual shape (length ca. 0.7× width) (Fig. 10a); margin between prostheca and mola of left mandible smooth (Fig. 10d)	***N.palawus* sp. nov.**

## ﻿Discussion

### ﻿Assignment to *Nigrobaetis*

For the assignment of the new species to *Nigrobaetis* we refer to Kluge (2022). Larval antennae of all new species are standing closely together, with a longitudinal keel between them (not figured); right mandibles always have a row of long, setae-like processes between prostheca and mola (Figs 2c, 10c, 16c, 22c, 29d, 35c); left mandibles usually have a row of minute denticles between prostheca and mola (Figs 2d, 22d, 29e, f, 35d), in one case the margin between prostheca and mola is bare (Fig. 10d), and in another case there is a tuft of medium, setae-like processes (Fig. 16d); labial palps segments II are without distomedial protuberance in all new species (Figs 4a, 12a, 18a, 24a, 31a, 37a); for *N.plures* sp. nov. and *N.suma* sp. nov., we observed the subimaginal gonostyli developing under cuticle of male last instar larvae and they are folded in the “*Nigrobaetis*-type” (Figs 6c, 26c). Further, as usually in *Nigrobaetis*, there is no real submarginal arc of setae dorsally on the labrum (Figs 2b, 10b, 16b, 22b, 29b, c), except for *N.kaliman* sp. nov., which has a short arc (Fig. 35b). The femoral patch is always absent.

### ﻿Morphological differences to *Nigrobaetis* species from Taiwan

Most species described from Taiwan have well developed hind protoptera (*N.candidus* (Kang & Yang, 1996), *N.mundus* (Chang & Yang, 1994), *N.taiwanensis* (Müller-Liebenau, 1985), *N.tatuensis* (Müller-Liebenau, 1985), *N.terminus* (Chang & Yang, 1994)), the exception is *N.facetus* (Chang & Yang, 1994) without hind protoptera (Kang et al. 1994; Kang and Yang 1996; Müller-Liebenau 1985). In this study, only *N.suma* sp. nov. has well developed hind protoptera, but it is distinguished from the Taiwanese species by the following combination of characters: abdominal pattern with terga I–VII and X brown, terga VIII and IX bright (Fig. 21a); seven pairs of tergalii (not six as *N.candidus*); labial palp segment III short, subquadrangular (Fig. 24a). *Nigrobaetisfacetus*, the only Taiwanese species without hind protoptera, is distinguished from the respective new species from Indonesia and the Philippines by the following characters: labrum rather wide with length ca. 0.6× maximum width, dorsally with a real submarginal arc of simple setae (the species in Indonesia and the Philippines have a less wide labrum with length to maximal width ca. 0.7–0.8×, and no submarginal arc, apart from *N.sumbensis* sp. nov. with a partial arc); the abdominal pattern of *N.sumbensis* sp. nov. (terga IV, VIII and IX bright) is different from the pattern of *N.facetus* (tergum V bright) (Fig. 15a; Kang et al. 1994: fig. 23).

### ﻿Morphology of eggs

Polygonal and partly rounded papillae-like structures as on the surface of the eggs of *N.plures* sp. nov. and *N.suma* sp. nov. (Figs 7, 40a) were also described from other *Nigrobaetis* species in Southeast Asia and the Palearctic region. It could be a character of the egg surface of a part of the *Nigrobaetis* species (see also Novikova and Kluge 1994): *N.mundus*, and *N.taiwanensis* from Taiwan, *N.niger* (Linnaeus, 1761), *N.digitatus* (Bengtsson, 1912), *N.bacillus* (Kluge, 1983), and *N.gracilis* (Bogoescu & Tabacaru, 1957) from the Palearctic (Kopelke and Müller-Liebenau 1981; Novikova and Kluge 1994; Kang et al. 1994) have this kind of surface. Other eggs of *Nigrobaetis* were described as having a surface without papillae-like structures, but also a polygonal pattern on the surface: e.g., *N.kogistani* Novikova & Kluge, 1994 from Eastern Palearctic (Novikova and Kluge 1994).

The eggs of *N.suma* sp. nov. in Fig. 27 could have a degraded surface. Therefore, it remains dubious to which of the two formerly mentioned groups of eggs they belong.

### ﻿Genetic distance

The genetic distance between three species of *Nigrobaetis* from Southeast Asia, for which we could obtain or download (GenBank) COI sequences (Indonesia, the Philippines, Thailand) is rather high (22%–23%, K2P), which is in line with the genetic distances found in *Labiobaetis* Novikova & Kluge, 1987 in Indonesia (11%–24%; Kaltenbach and Gattolliat 2019), Borneo (19%–25%; Kaltenbach and Gattolliat 2020), and the Philippines (15%–27%; Kaltenbach et al. 2020). Ball et al. (2005) reported a mean interspecific, congeneric distance of 18% for mayflies from the United States and Canada. The genetic distance between specimens of *N.suma* sp. nov. is very low as expected (0%–2%).

## Supplementary Material

XML Treatment for Nigrobaetis plures

XML Treatment for Nigrobaetis palawus

XML Treatment for Nigrobaetis sumbensis

XML Treatment for Nigrobaetis suma

XML Treatment for Nigrobaetis borneus

XML Treatment for Nigrobaetis kaliman

## ﻿Additional information

### Author ORCIDs

Thomas Kaltenbach https://orcid.org/0000-0001-8052-0388

Jean-Luc Gattolliat https://orcid.org/0000-0001-5873-5083

### Data availability

All of the data that support the findings of this study are available in the main text or Supplementary Information.

## References

[B1] Ball SL Hebert PDN Burian SK Webb JM (2005) Biological identifications of mayflies (Ephemeroptera) using DNA barcodes. Journal of the North American Benthological Society 24(3): 508–524. 10.1899/04-142.1

[B2] Barber-James HM Sartori M Gattolliat J-L Webb J (2013) World checklist of freshwater Ephemeroptera species. http://fada.biodiversity.be/group/show/35

[B3] Brown RM Diesmos AC (2010) Philippines, Biology. In: Gillespie RG Clague DA (Eds) Encyclopedia of Islands.University of California Press, Berkeley, Los Angeles, London, 723–732. 10.1525/9780520943728-170

[B4] Folmer O Black M Hoeh W Lutz R Vrijenhoek R (1994) DNA primers for amplification of mitochondrial cytochrome c oxidase subunit I from divers metazoan invertebrates. Molecular Marine Biology and Biotechnology 3: 294–299. http://www.mbari.org/staff/vrijen/PDFS/Folmer_94MMBB.pdf 7881515

[B5] Gattolliat J-L (2004) First reports of the genus *Nigrobaetis* Novikova & Kluge (Ephemeroptera: Baetidae) from Madagascar and La Réunion with observations on Afrotropical biogeography. Revue Suisse de Zoologie 111: 657–669. 10.5962/bhl.part.80259

[B6] Hall R (2010) Indonesia, Geology. In: Gillespie RG Clague DA (Eds) Encyclopedia of Islands.University of California Press, Berkeley, Los Angeles, London, 454–460. 10.1525/9780520943728-104

[B7] Hubbard MD (1995) Towards a standard methodology for the description of mayflies (Ephemeroptera). In: Corkum LD Ciborowski JJH (Eds) Current Directions in Research on Ephemeroptera.Canadian Scholar’s Press, Toronto, 361–369.

[B8] Jacobus LM Macadam CR Sartori M (2019) Mayflies (Ephemeroptera) and their contributions to ecosystem services. Insects 10(6): 1–26. 10.3390/insects10060170 PMC6628430 31207933

[B9] Kaltenbach T Gattolliat J-L (2019) The tremendous diversity of *Labiobaetis* Novikova & Kluge in Indonesia (Ephemeroptera, Baetidae). ZooKeys 895: 1–117. 10.3897/zookeys.895.38576 31844411 PMC6906171

[B10] Kaltenbach T Gattolliat J-L (2020) *Labiobaetis* Novikova & Kluge in Borneo (Ephemeroptera, Baetidae). ZooKeys 914: 43–79. 10.3897/zookeys.914.47067 32132855 PMC7046705

[B11] Kaltenbach T Garces JM Gattolliat J-L (2020) The success story of *Labiobaetis* Novikova & Kluge in the Philippines (Ephemeroptera, Baetidae), with description of 18 new species. ZooKeys 1002: 1–114. 10.3897/zookeys.1002.58017 33363429 PMC7746671

[B12] Kaltenbach T Mary N Gattolliat J-L (2021) The Baetidae (Ephemeroptera) of the Comoros and Mayotte. African Invertebrates 62(2): 427–463. 10.3897/afrinvertebr.62.70632

[B13] Kang S-C Yang C-T (1996) Two new species of *Baetis* Leach (Ephemeroptera: Baetidae) from Taiwan. Chinese Journal of Entomology 16: 61–66.

[B14] Kang C-H Chang H-C Yang C-T (1994) A revision of the genus *Baetis* in Taiwan (Ephemeroptera, Baetidae). Journal of Taiwan Museum 47: 9–44.

[B15] Kimura M (1980) A simple method for estimating evolutionary rates of base substitutions through comparative studies of nucleotide sequences. Journal of Molecular Evolution 16(2): 111–120. 10.1007/BF01731581 7463489

[B16] Kingston T (2010) Indonesia, Biology. In: Gillespie RG Clague DA (Eds) Encyclopedia of islands.University of California Press, Berkeley, Los Angeles, London, 446–453. 10.1525/9780520943728-103

[B17] Kluge NJ (2004) The phylogenetic system of Ephemeroptera. Academic Publishers, Dordrecht, 1–442. 10.1007/978-94-007-0872-3

[B18] Kluge NJ (2022) Ephemeroptera of the world. www.insecta.bio.spbu.ru/#Ephemeroptera [Retrieved November 2022]

[B19] Kopelke J-P Müller-Liebenau I (1981) Eistrukturen bei Ephemeroptera und deren Bedeutung bei der Aufstellung von Artengruppen am Beispiel der europäischen Arten der Gattung *Baetis* Leach, 1815. Teil III: *Buceratus*-, *atrebatinus*-, *niger*-, *gracilis*- und *muticus*-Gruppe (Ephemeroptera, Baetidae. Deutsche Entomologische Zeitschrift 28(1–3): 1–6. 10.1002/mmnd.19810280102

[B20] Kubendran T Balasubramanian C Selvakumar C Gattolliat J-L Sivaramakrishnan KG (2015) Contribution to the knowledge of *Tenuibaetis* Kang & Yang 1994, *Nigrobaetis* Novikova & Kluge, 1987 and *Labiobaetis* Novikova & Kluge, 1987 (Ephemeroptera: Baetidae) from the Western Ghats (India). Zootaxa 3957(2): 188–200. 10.11646/zootaxa.3957.2.3 26249065

[B21] Kumar S Stecher G Tamura K (2016) MEGA 7: Molecular evolutionary genetics analysis version 7.0 for bigger data sets. Molecular Biology and Evolution 33(7): 1870–1874. 10.1093/molbev/msw054 27004904 PMC8210823

[B22] Lugo-Ortiz CR de Moor FC (2000) *Nigrobaetis* Novikova & Kluge (Ephemeroptera: Baetidae): first record and new species from southern Africa, with reassignment of one northern African species. African Entomology 8: 69–73.

[B23] Müller-Liebenau I (1984) New genera and species of the family Baetidae from West-Malaysia (River Gombak) (Insecta: Ephemeroptera). Spixiana 7: 253–284.

[B24] Müller-Liebenau I (1985) Baetidae from Taiwan with remarks on *Baetiella* Ueno, 1931 (Ephemeroptera, Baetidae). Archiv für Hydrobiologie 104(1): 93–110. 10.1127/archiv-hydrobiol/104/1985/93

[B25] Novikova EA Kluge NJ (1994) Mayflies of the subgenus Nigrobaetis (Ephemeroptera, Baetidae, *Baetis*). Entomologicheskoe Obozrenie 73: 623–644. [In Russian]

[B26] Quek S-P (2010) Borneo. In: Gillespie RG Clague DA (Eds) Encyclopedia of Islands.University of California Press, Berkeley, Los Angeles, London, 111–116. 10.1525/9780520943728-029

[B27] Sanger F Nicklen S Coulson AR (1977) DNA sequencing with chain-terminating inhibitors. Proceedings of the National Academy of Sciences of the United States of America 74(12): 5463–5467. 10.1073/pnas.74.12.5463 271968 PMC431765

[B28] Sartori M Brittain JE (2015) Order Ephemeroptera. In: Thorp J Rogers DC (Eds) Ecology and General Biology: Thorp and Corvich’s Freshwater Invertebrates.Academic Press, 873–891. 10.1016/B978-0-12-385026-3.00034-6

[B29] Shorthouse DP (2010) SimpleMappr, an online tool to produce publication-quality point maps. [Retrieved from] https://www.simplemappr.net [accessed July 03, 2020]

[B30] Sivaruban T Srinivasan P Barathy S Isack R (2022) A new species of *Nigrobaetis* Novikova & Kluge, 1987 (Ephemeroptera, Baetidae) from Tamil Nadu, India. Zootaxa 5091(1): 182–190. 10.11646/zootaxa.5091.1.8 35391254

[B31] Tofilski A (2018) DKey software for editing and browsing dichotomous keys. ZooKeys 735: 131–140. 10.3897/zookeys.735.21412 PMC5904324 29674865

[B32] Vuataz L Sartori M Wagner A Monaghan MT (2011) Toward a DNA taxonomy of Alpine *Rhithrogena* (Ephemeroptera: Heptagenidae) using a mixed Yule-Coalescent Analysis of mitochondrial and nuclear DNA. PLoS ONE 6(5): 1–11. 10.1371/journal.pone.0019728 PMC3096624 21611178

